# Supplementary material for: Robust and flexible platform for directed evolution of yeast genetic switches
Source: Nat Commun. 2021 Mar 23;12:1846. doi: 10.1038/s41467-021-22134-y (PMC7988172; doi:10.1038/s41467-021-22134-y)
Supplement: Supplementary file 1 — Supplementary Information [file 41467_2021_22134_MOESM1_ESM.pdf]

Supplementary Information for

Robust and flexible platform for directed evolution of yeast genetic switches

Masahiro Tominaga, Kenta Nozaki, Daisuke Umeno, Jun Ishii, Akihiko Kondo

Contents

- Supplementary Figures 1–21
- Supplementary Tables 1–6
- Supplementary References

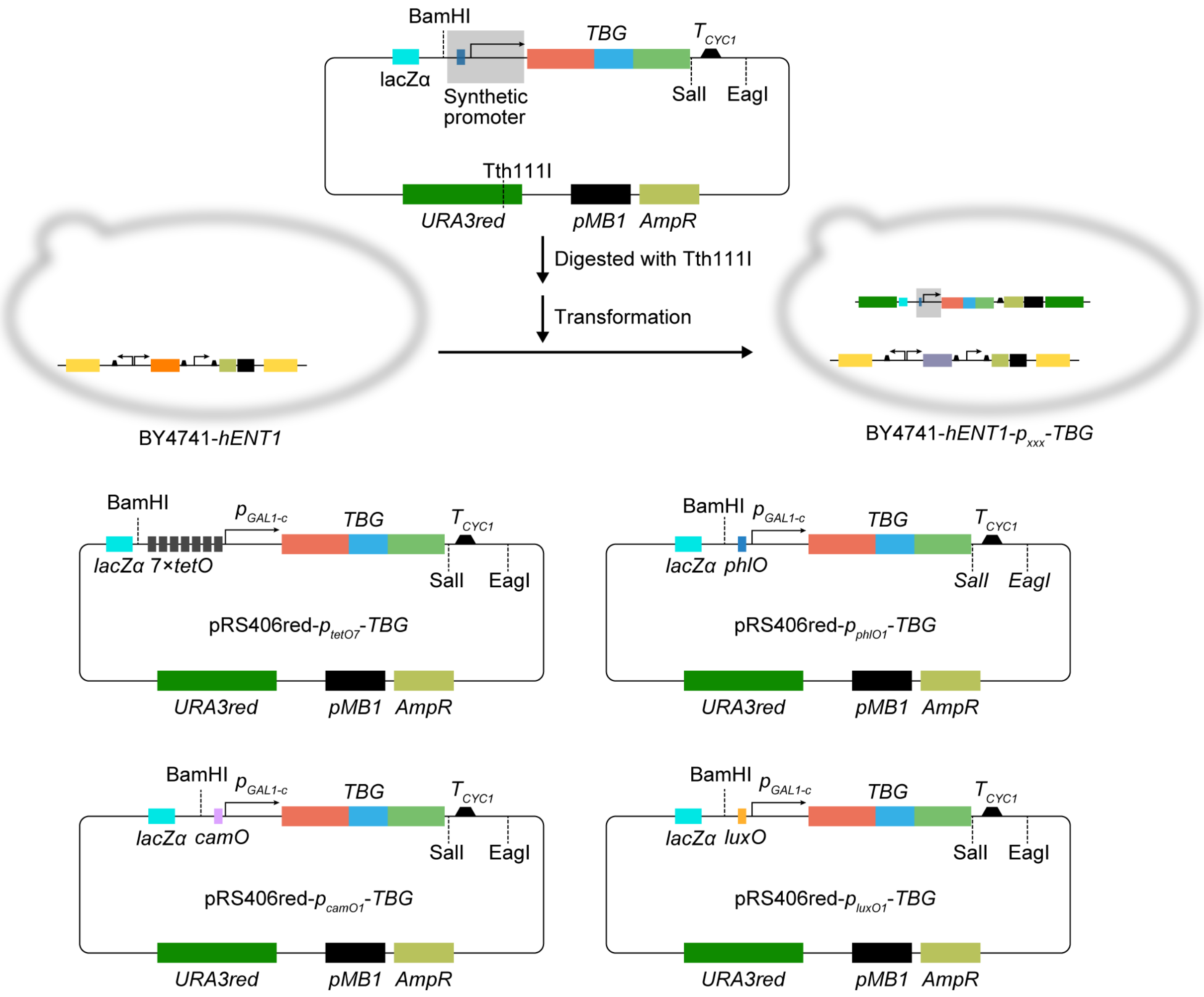

Supplementary Figure 1. Construction of *S. cerevisiae* strains with chromosomally integrated plasmids harbouring synthetic promoters driving expression of the *TBG* gene. The Tth111I-digested plasmids with different synthetic promoters (synPs) upstream of the *TBG* gene were integrated into the yeast genome. The plasmid maps of these plasmids are illustrated.

a

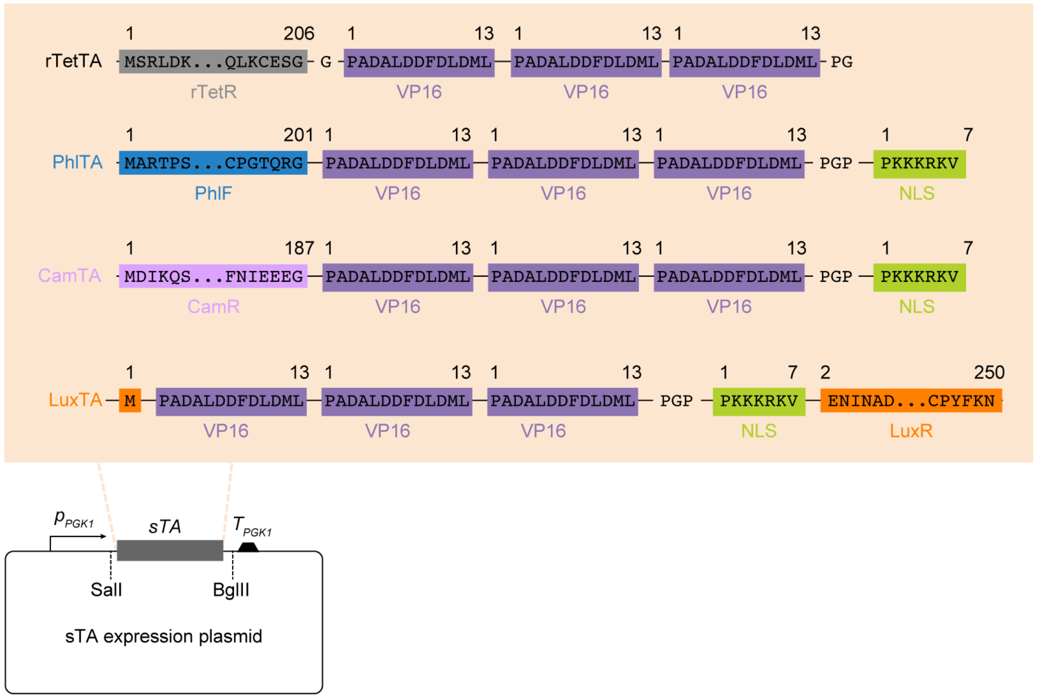

b

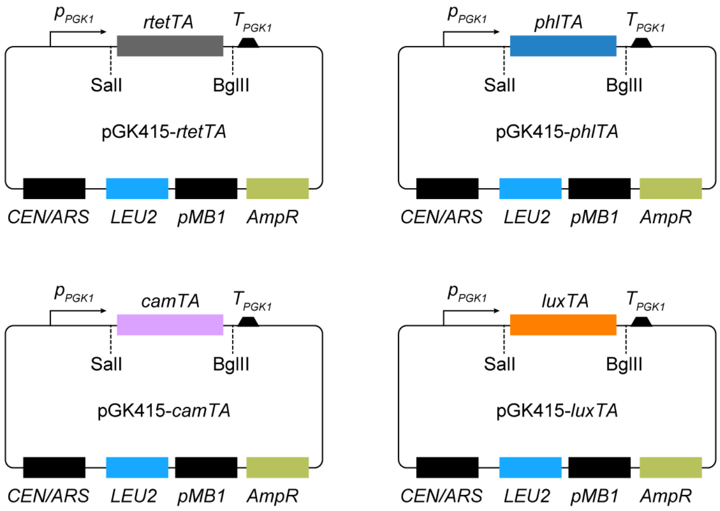

Supplementary Figure 2. Construction of plasmids encoding synthetic transcription activators (sTAs). (a) Partial amino acid sequences of sTAs. **(b)** Illustrations of the plasmids used for the expression of sTAs. *AmpR*, ampicillin resistant gene; *pMB1*, origin of replication in *E. coli*.

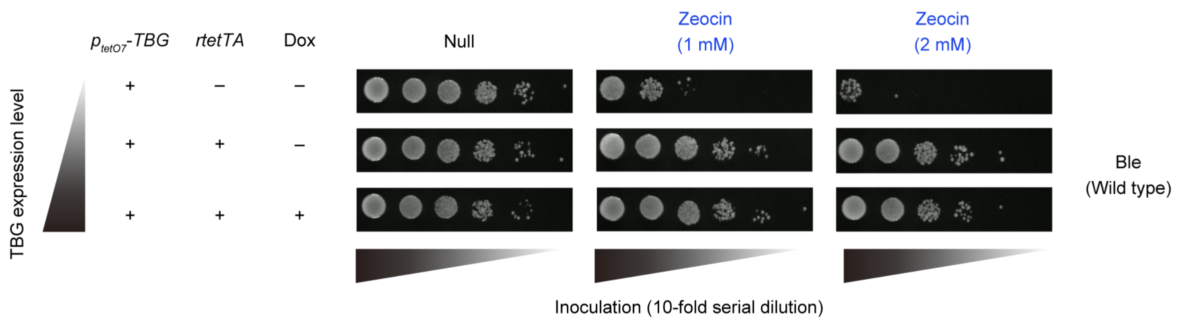

Supplementary Figure 3. TBG-expressing yeast harbouring a rTetTA-encoding episome are viable in the presence of Zeocin even in the absence of inducer (Dox). Yeast strains expressing the $TB_{WT}G$ protein (i.e., with wild-type Ble, lacking the D25A mutation) under control of p_{tetO7} were phenotypically assayed on selective medium. Cultures of each transformant were inoculated into selective liquid medium with Zeocin and incubated at 30 °C for 15 hours. Serial dilutions of the resulting cultures were spotted to SD medium supplemented with appropriate amino acids. Plates were photographed after 2 days of growth at 30 °C.

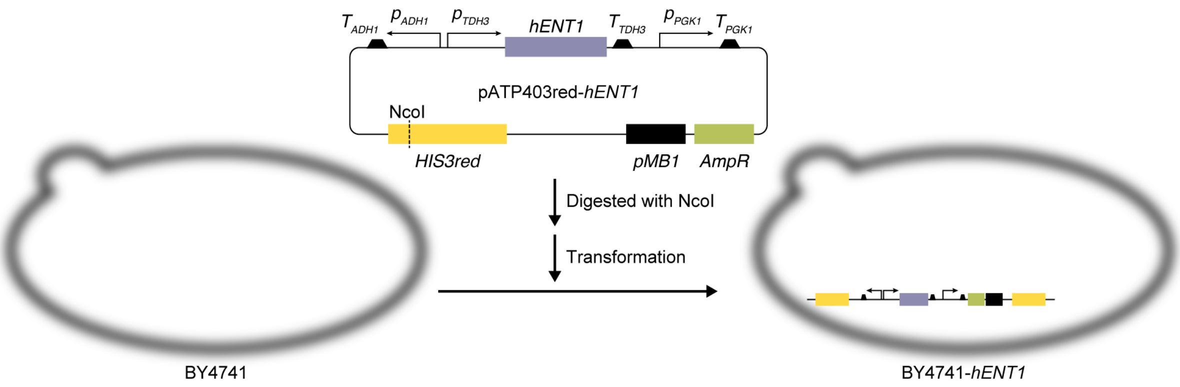

Supplementary Figure 4. Construction of *S. cerevisiae* strain harbouring hENT1-encoding plasmid. The NcoI-digested pATP403red-*hENT1* plasmid was integrated into the yeast genome.

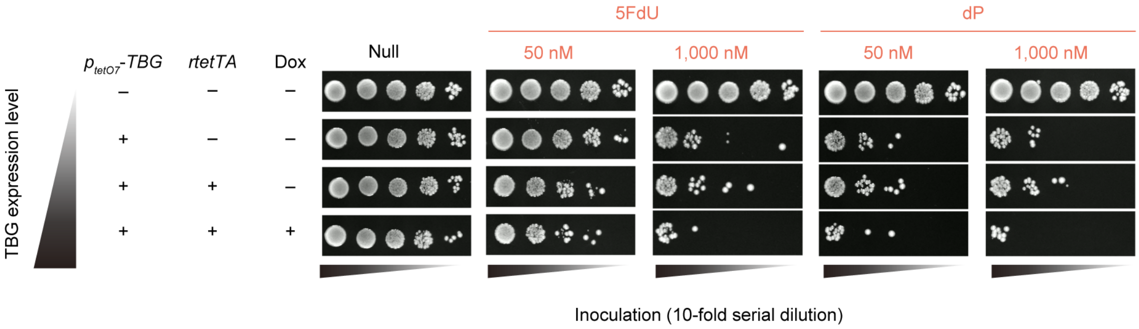

Supplementary Figure 5. Cells with leaky TBG expression do not survive OFF selection using the hsvTK/dP combination. 5FdU- or dP-sensitivity of yeast expressing the *TBG* gene at different strengths. Yeast strains expressing the TBG protein under the control of p_{tetO7} were phenotypically assayed on selective medium. Cultures of each transformant were inoculated into selective liquid medium with 5FdU or dP and incubated at 30 °C for 15 hours. Serial dilutions of the resulting cultures were spotted to SD medium supplemented with appropriate amino acids. Plates were photographed after 3 days of growth at 30 °C.

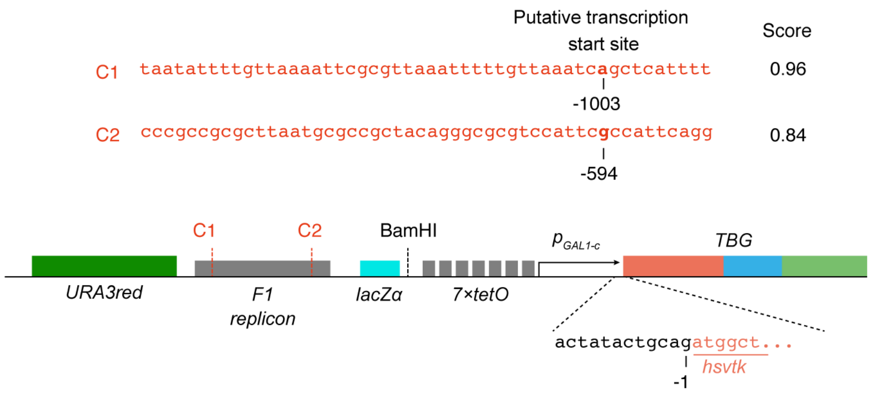

Supplementary Figure 6. Cryptic promoter prediction for sequences upstream of *p*$_{tetO7}$. The putative transcription start site and its arbitrary transcription efficiency (Score) were predicted using the Neural Network Promoter Prediction tool (https://www.fruitfly.org/seq_tools/promoter.html). Putative transcription start sites are shown as the distance from the start codon of *TBG*.

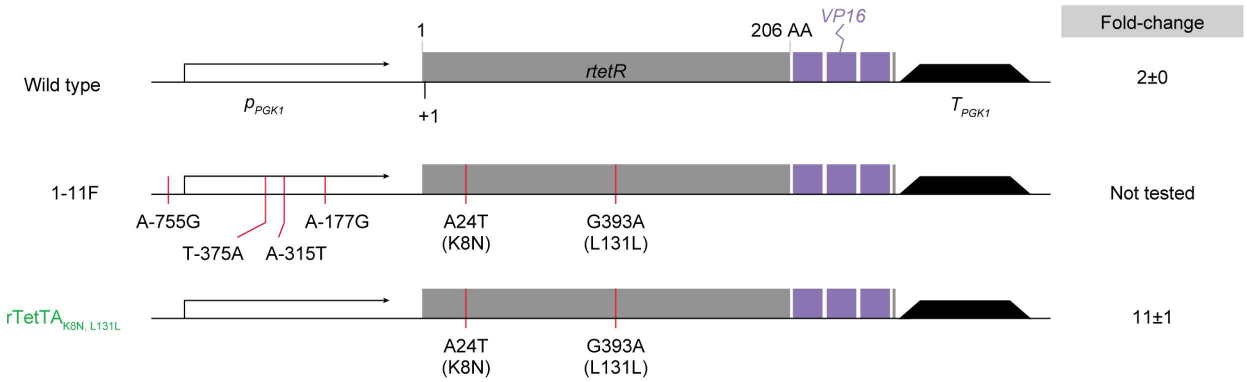

Supplementary Figure 7. Mutations found in the evolved Tet-ON switches. Nucleotide and amino acid (AA, shown in parentheses) mutations found in the rTetTA expression cassette are denoted with red lines. Fold-change was calculated from the data shown in **Fig. 3c** as the mean ± SD ratio of the fluorescence intensity in the presence and absence of 10 μg/mL Dox.

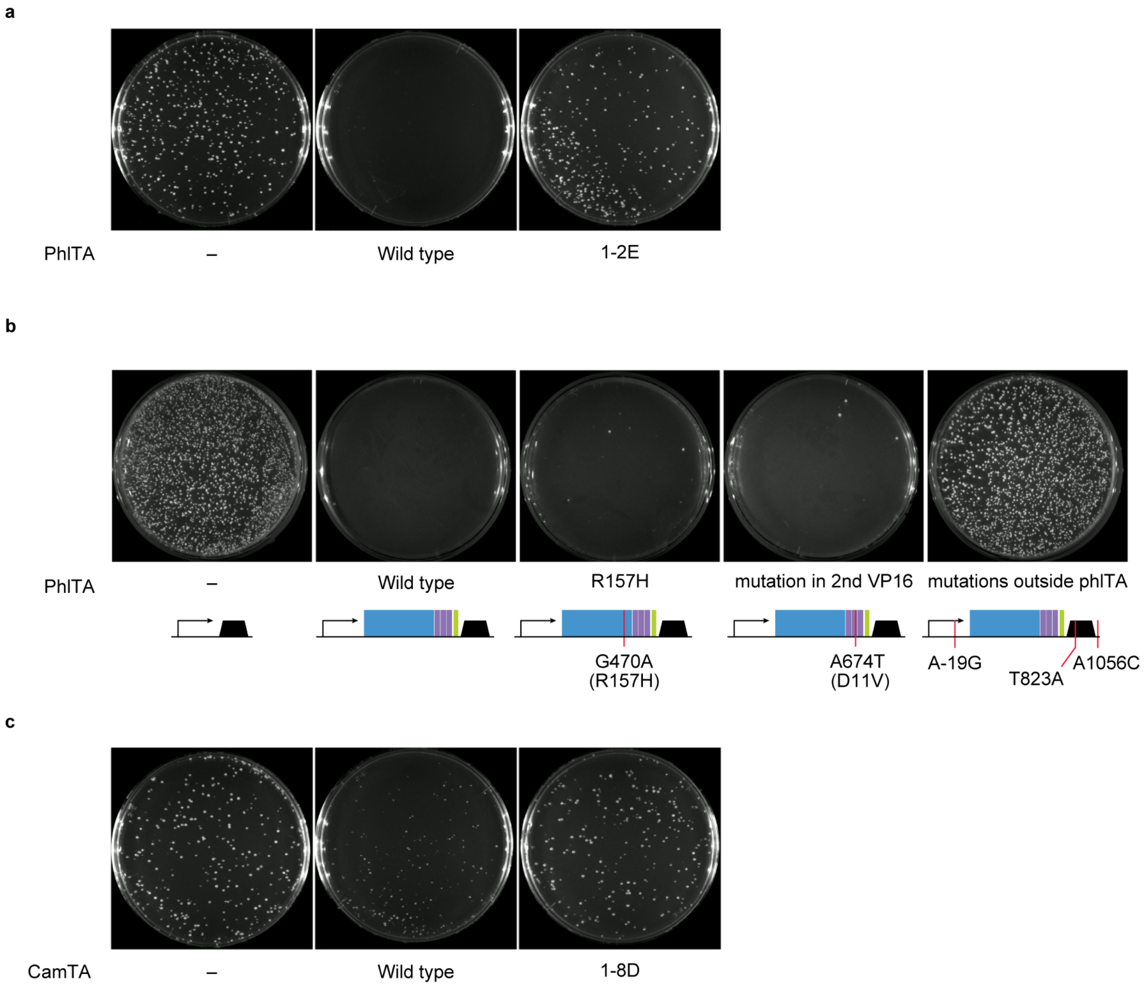

Supplementary Figure 8. Toxicity of synthetic transcription activator (sTA) overexpression. The same amount (400 ng) of plasmid encoding a wild-type or mutant sTA was used to transform *S. cerevisiae* strain (**a**, **b**) BY4741-*hENT1*-p_{phlO}-*TBG* or (**c**) BY4741-*hENT1*-p_{camO}-*TBG*. An aliquot (100 μL of 1 mL) of the transformation culture was inoculated onto SD selective agar, and the plates were incubated at 30 °C for 2 days.

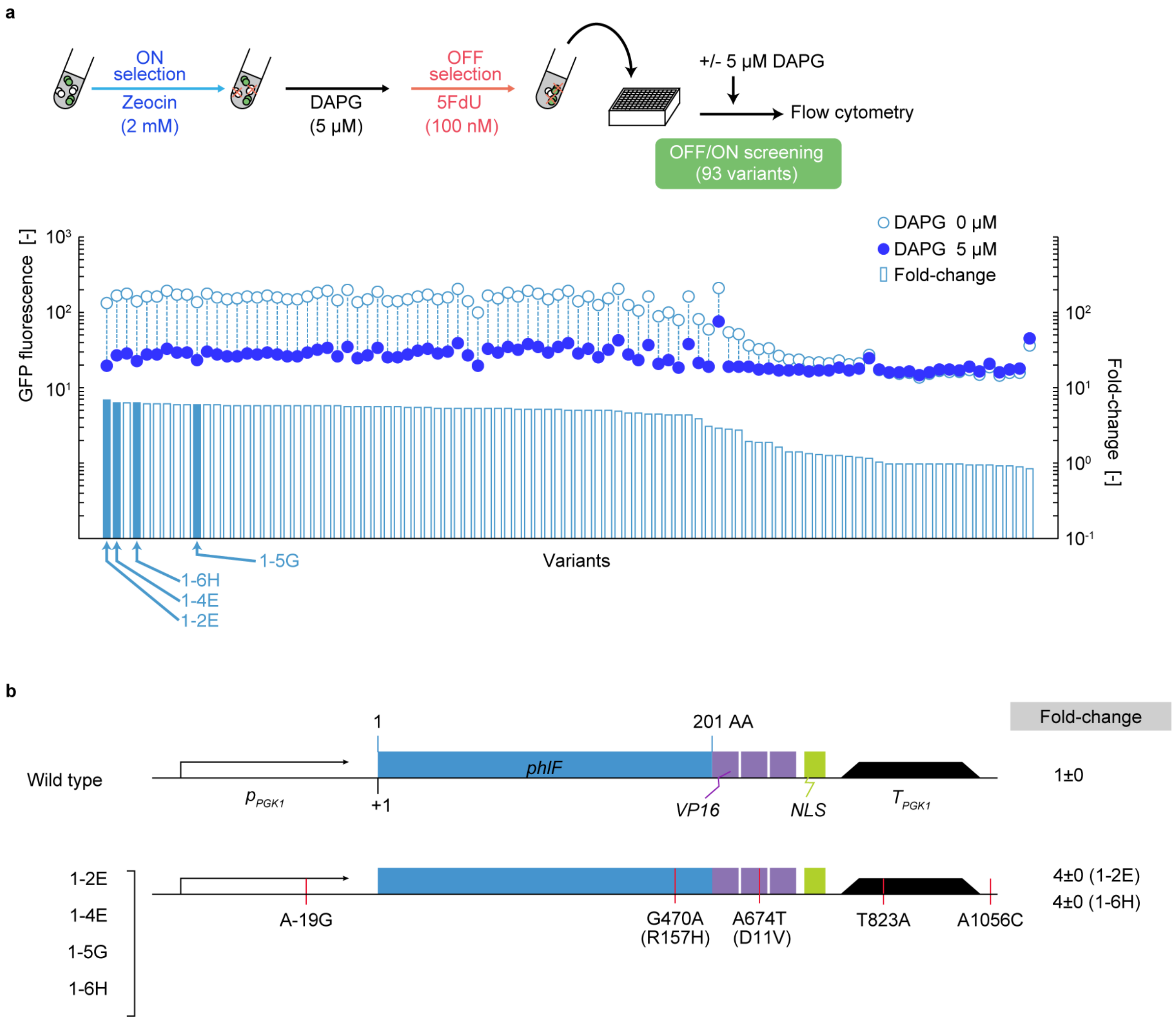

Supplementary Figure 9. Directed evolution to create a DAPG-OFF switch. (**A**) Selection procedure to isolate DAPG-OFF switches and to characterize randomly picked mutants from the selected library. First, cells were subjected to ON selection with Zeocin in the absence of inducers. Then, DAPG was added to the cell cultures to repress *TBG* gene expression, followed by the addition of 5FdU to eliminate cells with unwanted TBG expression. From the resultant cell populations, 93 clones were isolated and incubated in the presence or absence of DAPG for 24 hours at 30 °C. TBG-derived GFP fluorescence was measured for each sample using a flow cytometer. The bars represent the ratio of the signal in the ON/OFF state. The variants highlighted with arrows were subjected to dose-response testing **(Fig. 4b)** and/or **(b)** sequence analysis. Nucleotide and amino acid (AA, shown in parentheses) mutations found in the *phlTA* expression cassette are denoted with red lines. Fold-change was calculated from the data shown in **Fig. 4b** as the mean ± SD ratio of the fluorescence intensity in the absence and presence of 1 μM DAPG.

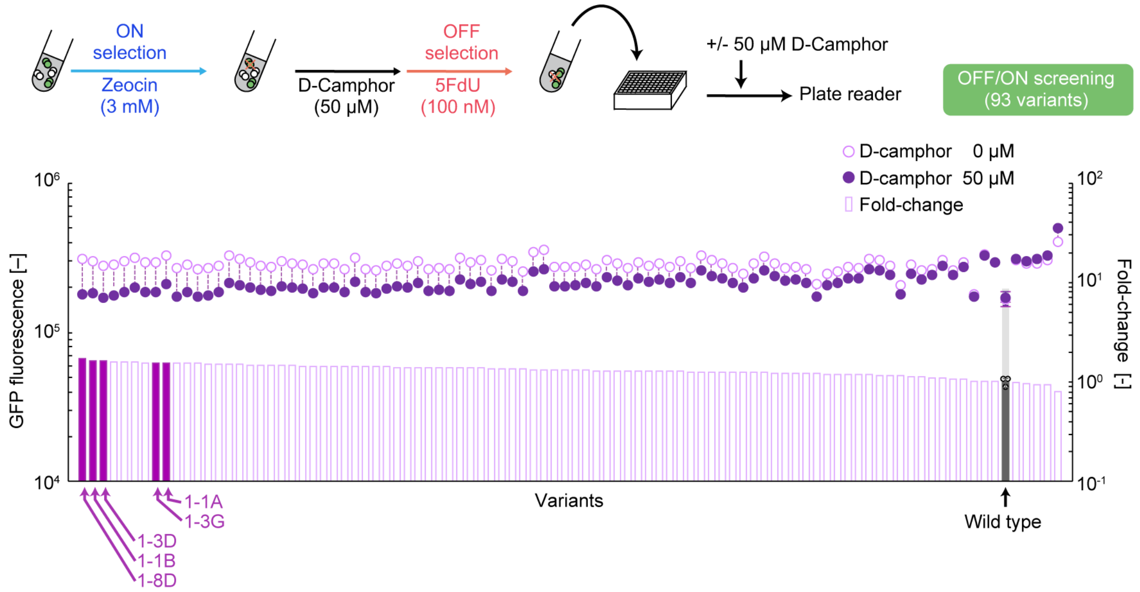

Supplementary Figure 10. Directed evolution to create a Camphor-OFF switch. Selection procedure to isolate Camphor-OFF switches and subsequent characterization of randomly picked mutants from the selected library. First, cells were subjected to ON selection with Zeocin in the absence of inducer. Then, D-camphor was added to the cell cultures to repress *TBG* gene expression, followed by the addition of 5FdU to eliminate cells with unwanted TBG expression. From the resultant cell populations, 93 clones were isolated and incubated in the presence or absence of D-camphor for 24 hours at 30 °C. TBG-derived GFP fluorescence was measured for each sample using a plate reader. The bars represent the ratio of the signal of the ON/OFF state (only for wild-type, N=3). Error bars represent the SD of three independent experiments. The variants highlighted with arrows were subjected to dose-response testing (**Fig. 4c**) and sequence analysis (**Supplementary Fig. 11**).

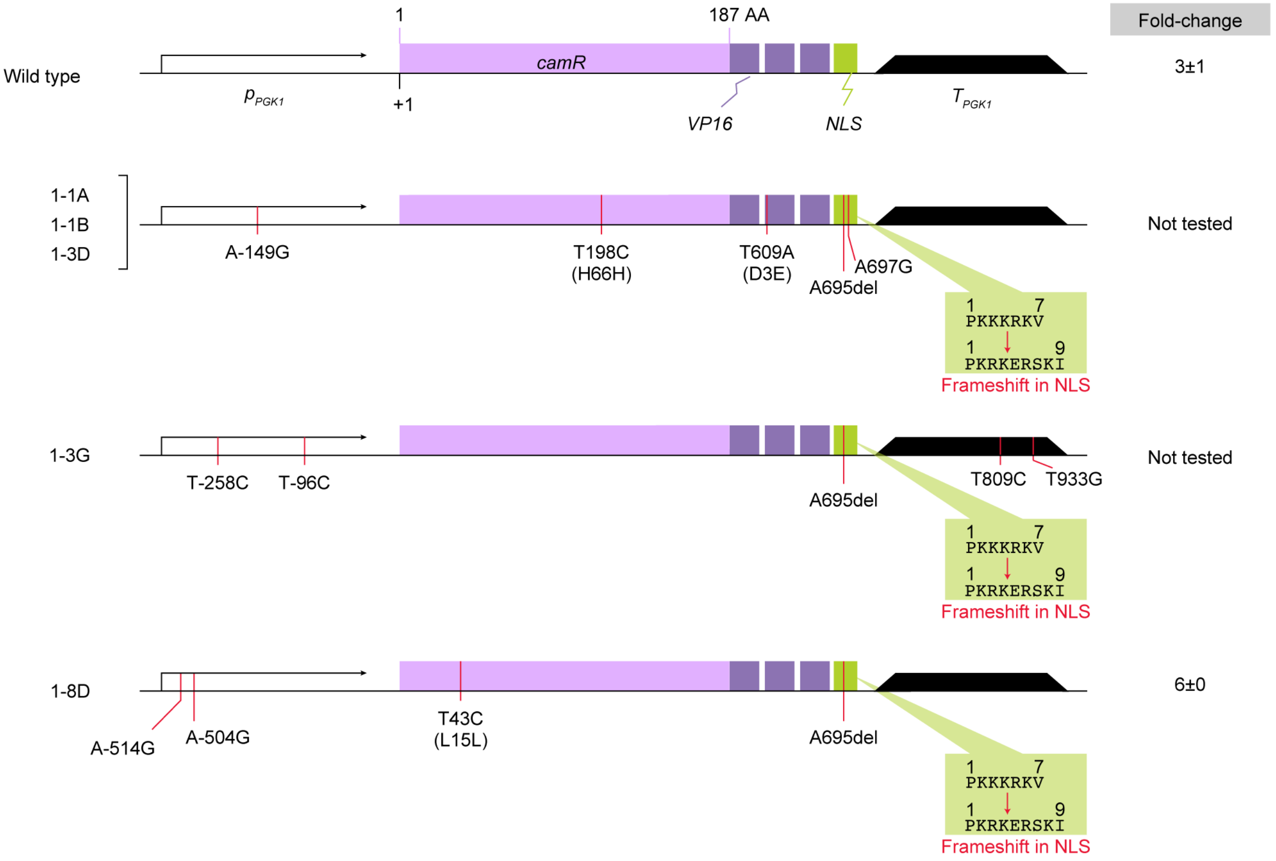

Supplementary Figure 11. Nucleotide and amino acid (AA, shown in parentheses) mutations found in the CamTA expression cassette of the evolved Camphor-OFF switches. Mutations found in the CamTA expression cassette are denoted with red lines. Fold-change was calculated from the data shown in **Fig. 4c** as the mean ± SD ratio of the fluorescence intensity in the absence and presence of 100 µM D-camphor.

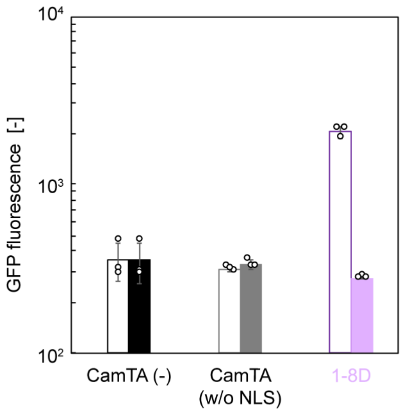

Supplementary Figure 12. Effect of the *nls* sequence on the switching behaviour of the Camphor-OFF switch. After growth for 24 hours in medium in the absence (open bar) or presence (filled bar) of 30 μM D-camphor, TBG-derived GFP fluorescence was measured. The data shown represent the mean ± standard deviation of three independent experiments.

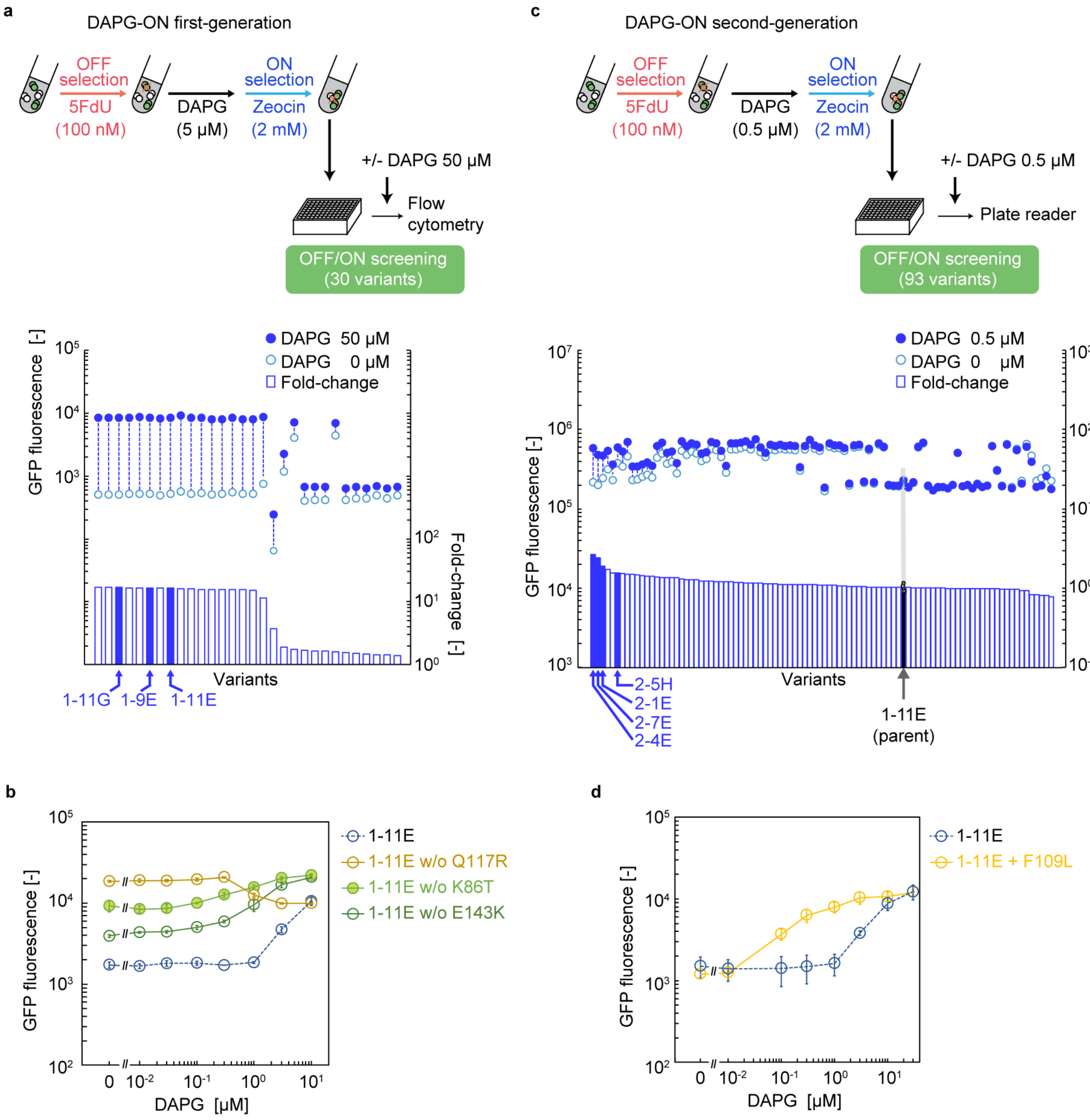

Supplementary Figure 13. Directed evolution to create a yeast DAPG-ON switch. Schemes of OFF/ON selection and subsequent screening from (**a**) first- and (**c**) second-generation libraries are illustrated. For the characterization of variants, TBG-derived GFP fluorescence was measured for each sample using a flow cytometer (first-generation library) or a plate reader (second-generation library) after growth at 30 °C for 24 hours in medium in the presence (filled circle) or absence (open circle) of inducer. The bars represent the ratio of the signal of the ON/OFF state (only for 1-11E variant in (c), N=3). The variants highlighted with arrows were subjected to detailed analysis (**Fig. 4d**) and sequencing (**Supplementary Fig. 14**). These analyses identified mutations that yielded PhlTA with reversed response (rPhlTA) to DAPG (**b**) and rPhlTA sensitized to DAPG (**d**). Error bars represent the SD of three independent experiments.

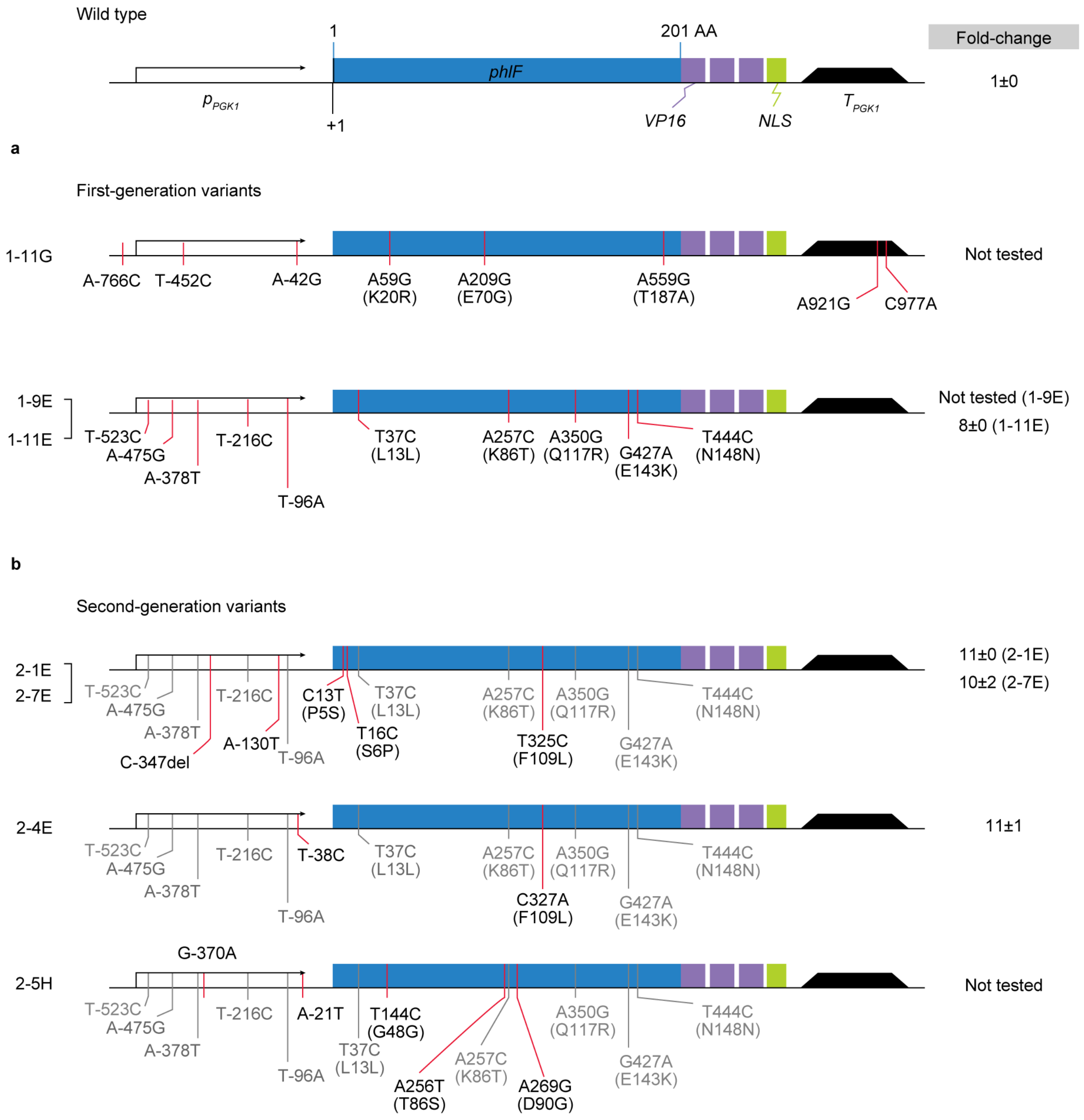

Supplementary Figure 14. Nucleotide and amino acid (AA, shown in parentheses) mutations found in the rPhlTA expression cassette of the evolved DAPG-ON switches. Mutations found in the **(a)** first- and **(b)** second-generation rPhlTA expression cassette are denoted with red lines, excluding the second-generation mutants whose mutations also were found in the parental (1-11E) mutant, which are denoted in grey. Fold-change was calculated from the data shown in **Fig. 4d** as the mean ± SD ratio of the fluorescence intensity in the presence and absence of 10 µM DAPG.

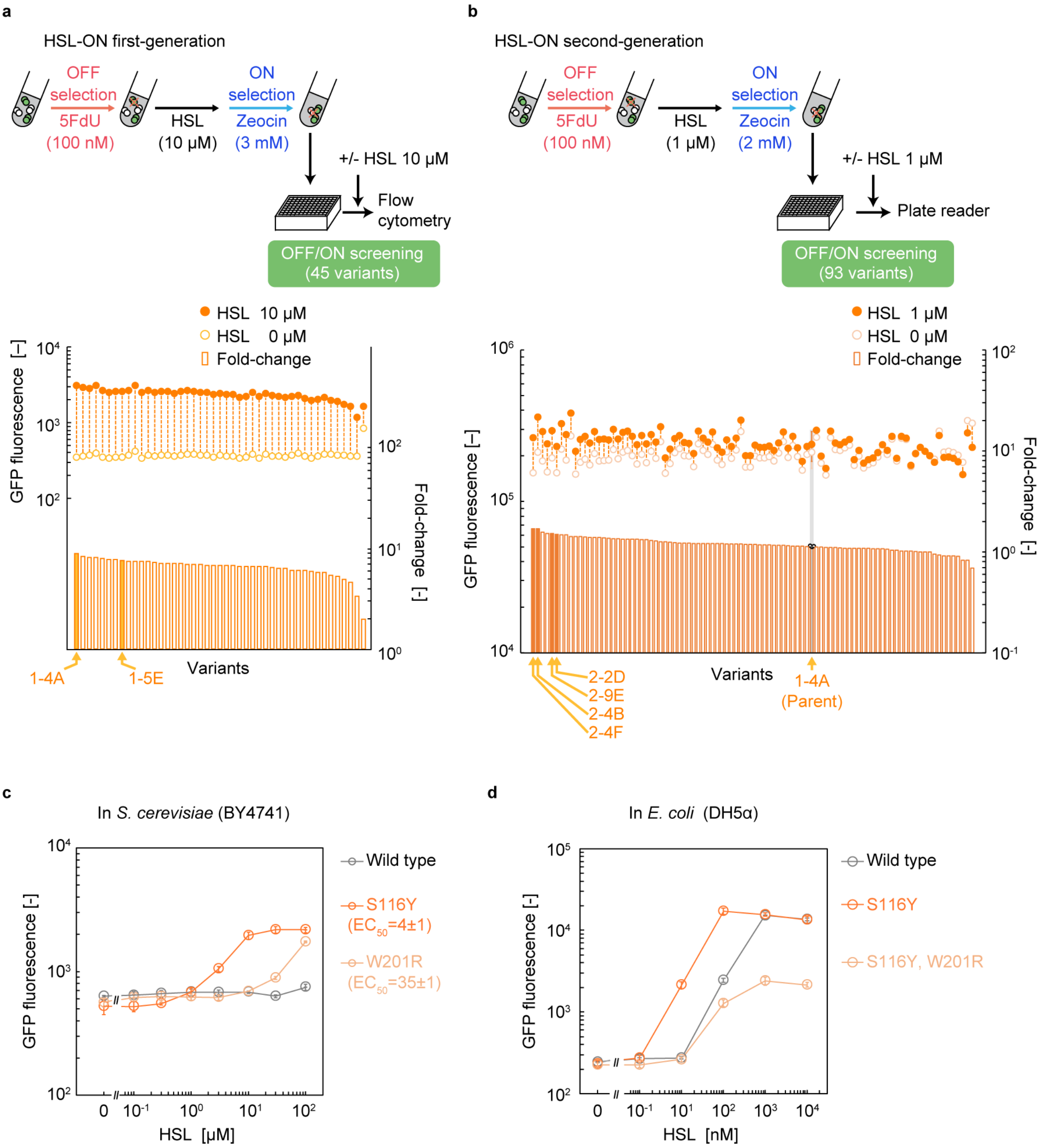

Supplementary Figure 15. Directed evolution to create HSL-ON switch. Schemes of OFF/ON selection and subsequent screening from (**a**) first- and (**b**) second-generation libraries are illustrated. For the characterization of variants, TBG-derived GFP fluorescence was measured for each sample using a flow cytometer (first-generation library) or a plate reader (second-generation library) after growth for 24 hours at 30 °C in medium in the presence (filled circle) or absence (open circle) of inducer. The bars represent the ratio of the signal of the ON/OFF state (only for 1-4A variant in (B), N=3).. The variants highlighted with arrows were subjected to dose-response testing (**Fig. 4f**) and/or sequence analysis (**Supplementary Fig. 16**). The effect of each mutation (S116Y and W201R) was evaluated in (**c**) *S. cerevisiae* (BY4741-*hENT1*-p_{luxO1}-*TBG*) and (**d**) *E. coli* (DH5α). Error bars represent the SD of three independent experiments.

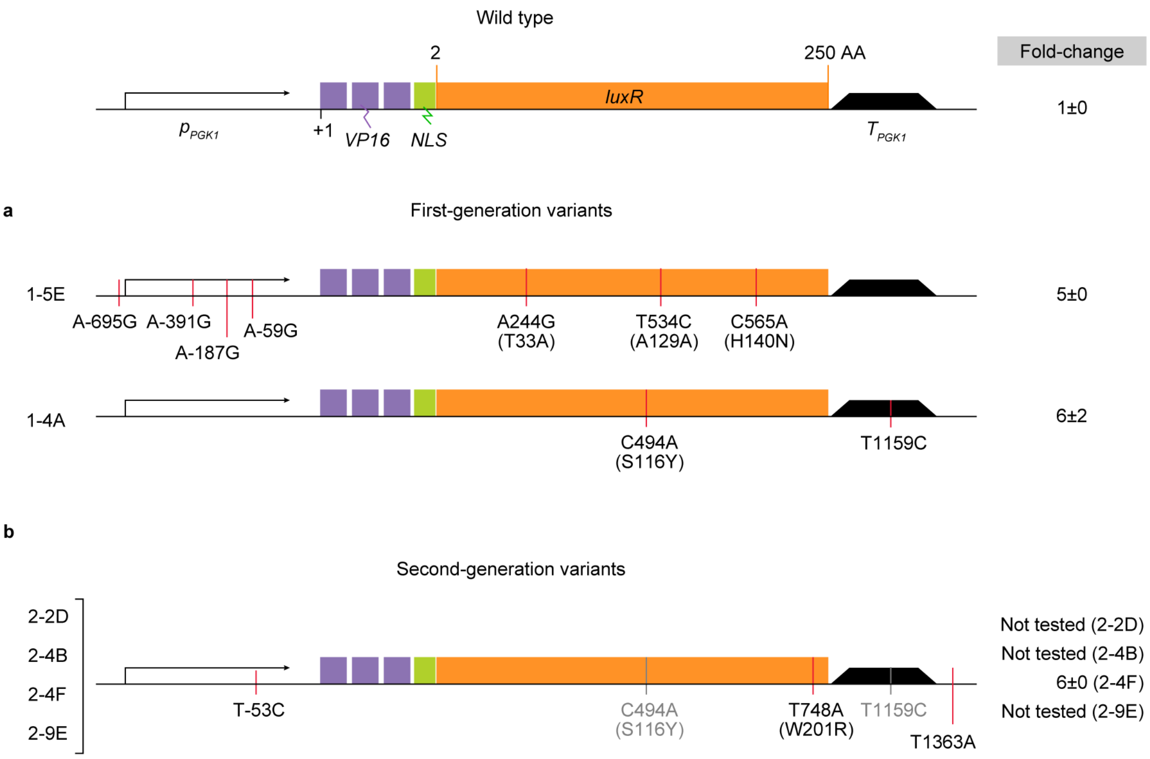

Supplementary Figure 16. Nucleotide and amino acid (AA, shown in parentheses) mutations found in the LuxTA expression cassette of the evolved HSL-ON switches. Mutations found in the **(a)** first- and **(b)** second-generation LuxTA expression cassette are denoted by red lines, excluding the second-generation mutants whose mutations also were found in the parental (1-4A) mutant, which are denoted in grey. Fold-change was calculated from the data shown in **Fig. 4f** as the mean ± SD ratio of the fluorescence intensity in the presence and absence of 100 μM HSL (3 μM for 2-4F).

a

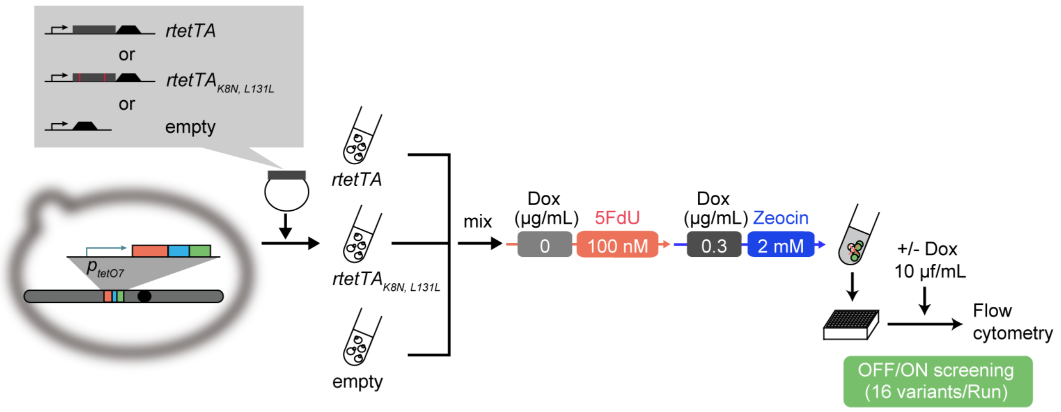

b

Relative abundance of each variant [%]						
variant	Run-1		Run-2		Run-3	
	Before	After	Before	After	Before	After
rtetA$_{K8N,\ L131L}$	0.047	6.25 (1/16)	0.038	N. D. (0/16)	0.053	6.25 (1/16)
rtetA	48.1	81.3 (13/16)	42.7	81.3 (13/16)	44.7	25.0 (4/16)
empty	51.9	12.5 (2/16)	57.2	18.8 (3/16)	55.2	68.8 (10/16)
Enrichment factor for *rtetA*$_{K8N,\ L131L}$	133		N. D.		118	

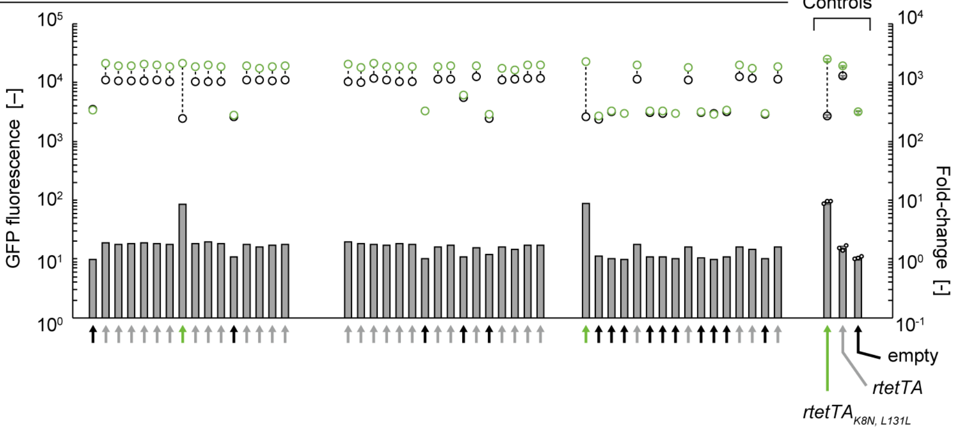

Supplementary Figure 17. Enrichment for the mutant Tet-ON switch (Tet-ON$_{K8N,\ L131L}$) from mock switch library. (**a**) Yeast cells harboring plasmid with wild-type Tet-ON or Tet-ON$_{K8N,\ L131L}$, and empty vector was mixed at a ratio of approximately 1:10^{-3}:1. The actual abundance of Tet-ON$_{K8N,\ L131L}$ was evaluated by counting the colony forming units of each yeast culture. The resultant cell culture was subjected to OFF/ON selections with the same condition of Run-11 as described in **Figure 3**. The same selection experiments were independently performed three times. Colonies were isolated from the resultant three cell populations and the Dox-induced GFP expression was evaluated using flow-cytometry (**b**). The Tet-ON$_{K8N,\ L131}$ variants in the selected cell pools were identified by a Dox-inducible GFP expression pattern comparable to that of the Tet-ON$_{K8N,\ L131}$ control. The bars represent the ratio of the signal of the ON/OFF state (only for controls, N=3. Error bars represent the SD of three independent experiments). Variants identified as cells harboring plasmid expressing rtetTA$_{K8N,\ L131L}$ or wild-type rtetTA, and empty vector are denoted by green, grey, and black arrows, respectively.

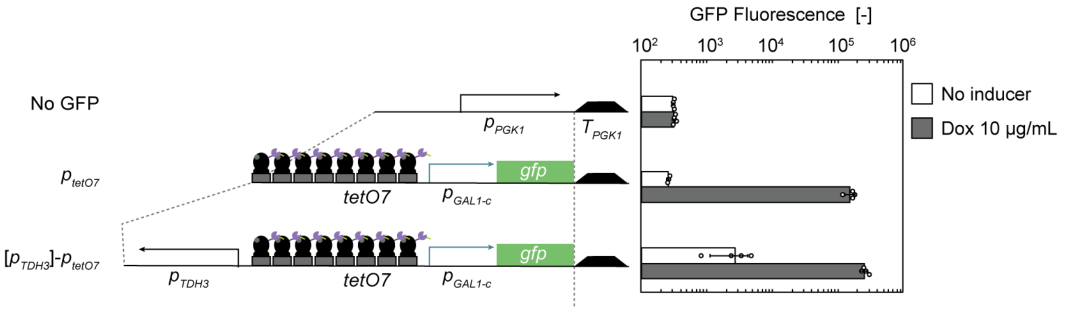

Supplementary Figure 18. **Context-dependent leakiness of *p*$_{tetO7}$**. Plasmids with *gfp* gene downstream of *p*$_{tetO7}$ with and without the upstream reverse-oriented *p*$_{TDH3}$ was used to transform the same yeast strain used in **Figure 5a,** which harbours the plasmids expressing an sTA via the Tet-ON$_{K8N,\ L131L}$, DAPG-ON$_{2\text{-}1E}$, and HSL-ON$_{2\text{-}4F}$ switches. GFP expression was measured using flow cytometry. The data shown represent the mean ± standard deviation of four independent experiments (only for *p*$_{tetO7}$ without Dox, N=3).

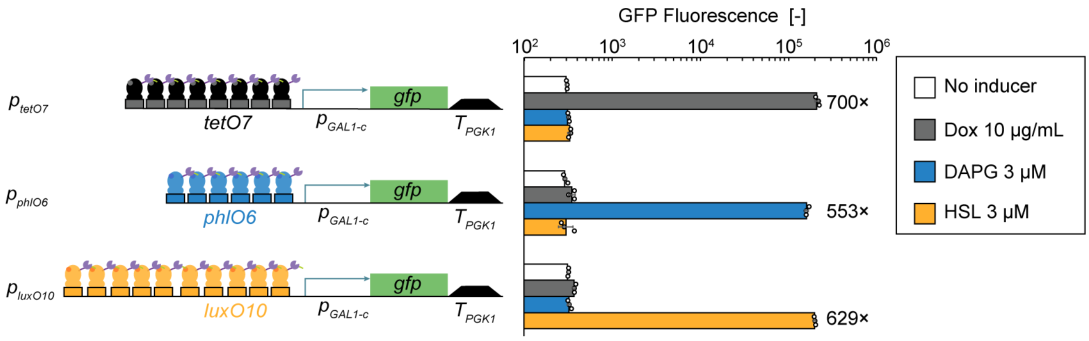

Supplementary Figure 19. Orthogonal GFP expression controlled by each synP (*p_{phlO6}*, *p_{tetO7}*, and *p_{luxO10}*). Each of the plasmids expressing an sTA via the Tet-$ON_{K8N,\ L131L}$, DAPG-$ON_{2\text{-}1E}$, and HSL-$ON_{2\text{-}4F}$ switches was integrated into the chromosome as depicted in **Figure 5a**. The *gfp* gene downstream of each synP (p_{tetO7}, p_{phlO6}, and p_{luxO10}) is expressed only in the presence of the corresponding inducer (Dox, DAPG, and HSL, respectively). Orthogonal GFP expression control using Dox, DAPG, and HSL was measured using flow cytometry. The data shown represent the mean ± standard deviation of three independent experiments.

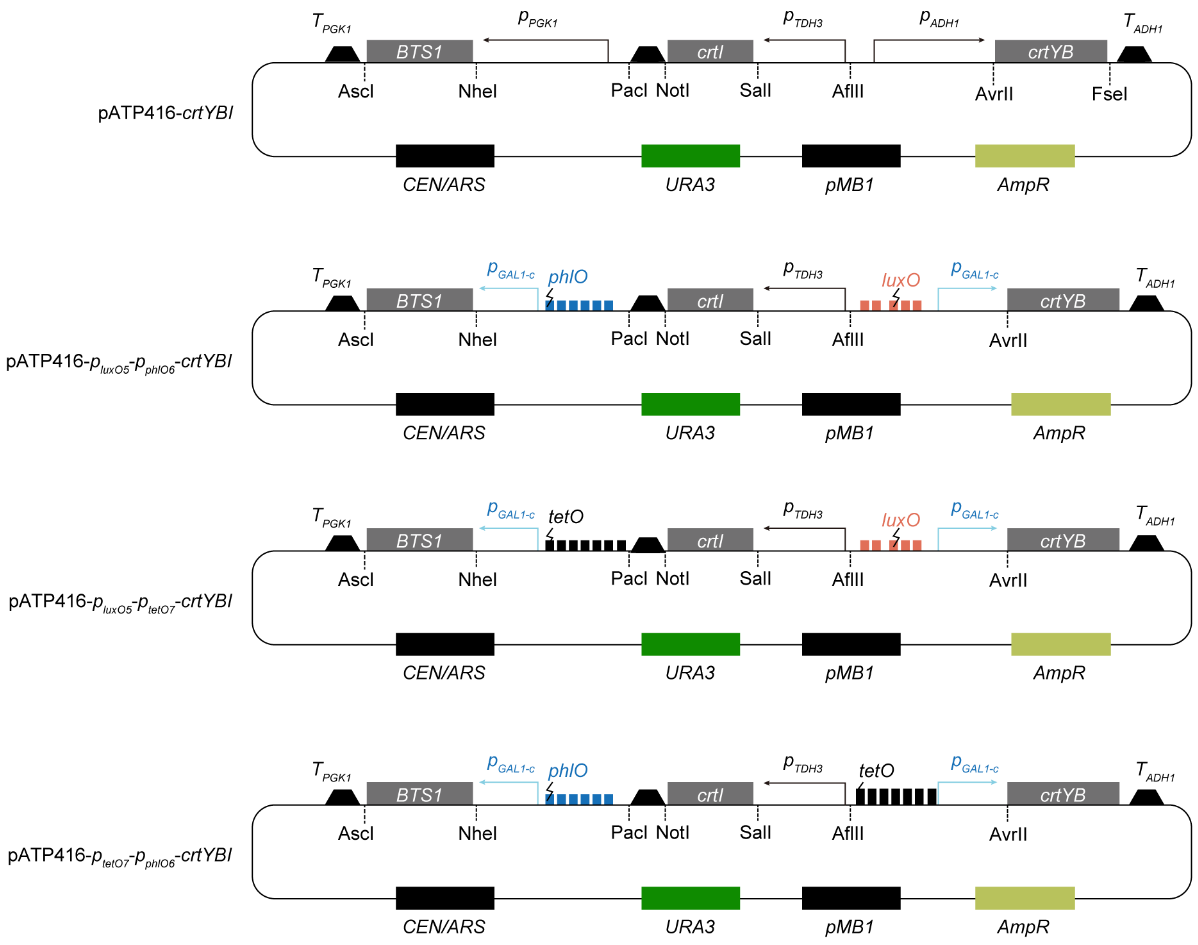

Supplementary Figure 20. Plasmid maps for the regulation of carotenoid production used in Fig. 5d.

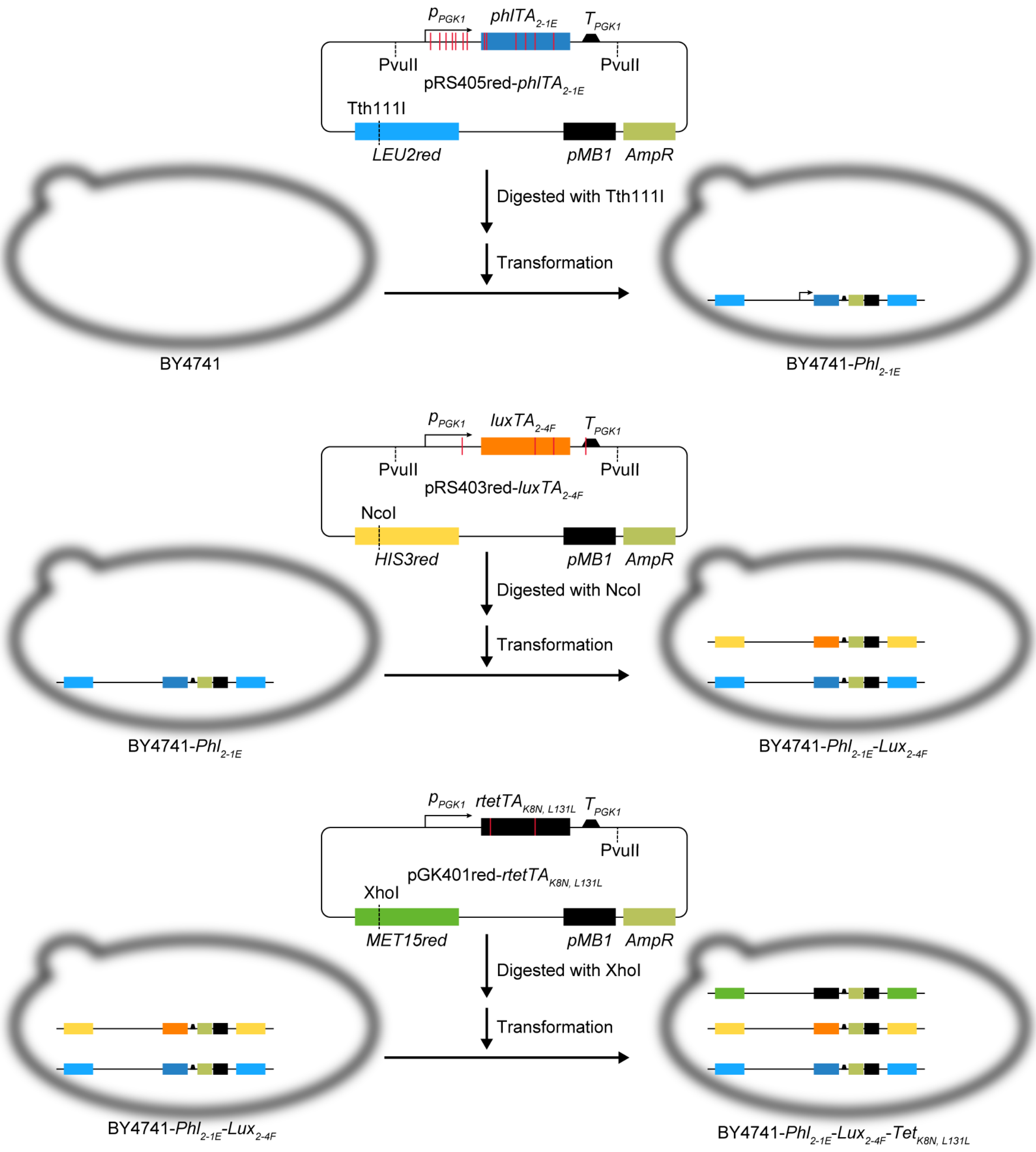

Supplementary Figure 21. Construction scheme for *S. cerevisiae* strains BY4741-*Phl*$_{2\text{-}1E}$, BY4741-*Phl*$_{2\text{-}1E}$-*Lux*$_{2\text{-}4F}$, and BY4741-*Phl*$_{2\text{-}1E}$-*Lux*$_{2\text{-}4F}$-*Tet*$_{K8N,\ L131L}$ used for orthogonal and simultaneous gene induction in Figure 5 and Supplementary Figure 19.

Supplementary Table 1. The frequency of OFF-selection escapees.

Strain + plasmid	N	Frequency of 5FdU^r colony [-]	Frequency of dP^r colony [-]
BY4741-*hENT1*-p_{tetO7}-*TBG*+ pGK415-*rTetTA*	1	4×10^{-4}	4×10^{-4}
	2	2×10^{-3}	1×10^{-3}
	3	8×10^{-4}	5×10^{-4}
	Ave ± SD	$1\times10^{-3}\pm7\times10^{-4}$	$8\times10^{-4}\pm5\times10^{-4}$
BY4741-*hENT1*-p_{tetO7}-*TBG/hENT1*+pGK415-*rTetTA*	1	8×10^{-6}	6×10^{-6}
	2	2×10^{-6}	2×10^{-6}
	3	$<4\times10^{-7}$	$<4\times10^{-7}$

5FdU^r: 5FdU resistant, dP^r: dP resistant

Supplementary Table 2. *E. coli* and yeast strains used in this study.

Strain name	Relevant features	Source
DH5α	-	TOYOBO
BY4741	*MAT***a** *his3Δ1 ura3Δ0 leu2Δ0 met15Δ*	(1)
BY4741-*hENT1*	BY4741/pATP403red-*hENT1*	This study
BY4741-*hENT1*-p_{tetO7}-*TBG*	BY4741-hENT1/pATP406red-p_{tetO7}-*TBG*	This study
BY4741-*hENT1*-p_{tetO7}-*TBG/hENT1*	BY4741-*hENT1*-p_{tetO7}-*TBG*/pGK401red-hENT1	This study
BY4741-*hENT1*-p_{phlO1}-*TBG*	BY4741-hENT1/pATP406red-p_{phlO1}-*TBG*	This study
BY4741-*hENT1*-p_{camO1}-*TBG*	BY4741-hENT1/pATP406red-p_{camO1}-*TBG*	This study
BY4741-*hENT1*-p_{luxO1}-*TBG*	BY4741-hENT1/pATP406red-p_{luxO1}-*TBG*	This study
BY4741-$Phl_{2\text{-}1E}$	BY4741/pRS405red-$phlTA_{2\text{-}1E}$	This study
BY4741-$Phl_{2\text{-}1E}$-$Lux_{2\text{-}4F}$	BY4741-$phl_{2\text{-}1E}$ /pRS403red-$luxTA_{2\text{-}4F}$	This study
BY4741-$Phl_{2\text{-}1E}$-$Lux_{2\text{-}4F}$-$Tet_{K8N,\ L131L}$	BY4741-$phl_{2\text{-}1E}$-$Lux_{2\text{-}4F}$ /pRS401red-$tetTA_{K8N,\ L131L}$	This study

Supplementary Table 3. Plasmids used in this study.

Plasmid name	Relevant features	Source
pACYCDuet^{TM} -1	Expression vector for *E. coli*	Novagen
pGK415	Expression vector containing p_{PGK1}, T_{PGK1}, the *CEN/ARS* replication origin, and the *LEU2* marker	(2)
pRS403	Integration vector (without yeast replication origin) with the *HIS3* marker	ATCC
pRS405	Integration vector (without yeast replication origin) with the *LEU2* marker	ATCC
pRS406	Integration vector (without yeast replication origin) with the *URA3* marker	ATCC
pRS416	Expression vector containing the *CEN/ARS* replication origin and *URA3* marker	ATCC
pATP403	Genome integration vector containing three sets of promoters/terminators (p_{ADH1}/T_{ADH1}, p_{TDH3}/T_{TDH3}, and p_{PGK1}/T_{PGK1},) and the *HIS3* marker	(3)
pATP406	Genome integration vector containing three sets of promoters/terminators (p_{ADH1}/T_{ADH1}, p_{TDH3}/T_{TDH3}, and p_{PGK1}/T_{PGK1},) and the *URA3* marker	(3)
pATP426	Expression vector containing three sets of promoters/terminators (p_{ADH1}/T_{ADH1}, p_{TDH3}/T_{TDH3}, and p_{PGK1}/T_{PGK1},) and the *URA3* marker	(3)
pATP403red	Modified version of pATP403 with an extended *HIS3* marker	This study
pRS403red	Modified version of pRS403 with an extended *HIS3* marker	This study
pRS405red	Modified version of pRS405 with an extended *LEU2* marker	This study
pRS406red	Modified version of pRS406 with an extended *URA3* marker	This study
pFS181	Plasmid vector used as a template for amplifying *hENT1*.	(4)
pATP403red-*hENT1*	pATP403red vector with *hENT1* downstream of p_{TDH3}.	This study

pGK401red-*hENT1*	pRS401red vector with *hENT1* downstream *of* p_{PGK1}	pGK401red-hENT1
pTRE-tight	Template plasmid for PCR amplification of p_{tetO7}	Clontech Laboratories
pBT3-C	Template plasmid for PCR amplification of *CYC1* terminator	MoBiTec
pRS406red-p_{tetO7} -*TBG*	pRS406red vector with p_{tetO7} and downstream *TBG*	This study
pRS406red-p_{phlO1} -*TBG*	pRS406red vector with p_{phlO1} and downstream *TBG*	This study
pRS406red-p_{camO1} -*TBG*	pRS406red vector with p_{camO1} and downstream *TBG*	This study
pRS406red-p_{luxO1} -*TBG*	pRS406red vector with p_{luxO1} and downstream *TBG*	This study
pGK416-*ymUkG1*	pGK416 vector with *ymUkG1* (gene encoding monomeric Umikinoko-Green1, codon-optimized for *S. cerevisiae*)	(5)
pCMV-Tet3G	Template plasmid encoding rTetTA (rtTA2^{S}-M2)	Clontech Laboratories
pGK415-*rTetTA*	pGK415 vector with *rtetTA* downstream of p_{PGK1}	This study
pGK415-*phlTA*	pGK415 vector with *phlTA* downstream of p_{PGK1}	This study
pGK415-*camTA*	pGK415 vector with *camTA* downstream of p_{PGK1}	This study
pGK415-*camTA (-nls)*	pGK415 vector with *camTA* (lacking *nls)* downstream of p_{PGK1}	This study
pGK415-*luxTA*	pGK415 vector with *luxTA* downstream of p_{PGK1}	This study
pRS403red-$luxTA_{2\text{-}4F}$	pATP403red vector with the mutant expression cassette for *luxTA* (2-4F)	This study
pRS405red-$phlTA_{2\text{-}1E}$	pRS405red vector with the mutant expression cassette for *phlTA* (2-1E)	This study
pGK415-$rtetTA_{K8N,\ L131L}$	pGK415 vector with $rtetTA_{K8N,\ L131L}$	This study
pRS401red-$rtetTA_{K8N,\ L131L}$	pRS401red vector with $rtetTA_{K8N,\ L131L}$	This study
pGK416m-p_{tetO7}-*ymUkG1*	p_{tetO7} cloned upstream of *ymUkG1* gene in pGK416-*ymUkG1* (via NsiI and NheI sites)	This study
pGK416m-p_{phlO6}-*ymUkG1*	p_{phlO6} cloned upstream of *ymUkG1* gene in	This study

	pGK416-*ymUkG1* (via NsiI and NheI sites)	
pGK416m-p_{luxO5}-*ymUkG1*	p_{luxO5} was cloned upstream of *ymUkG1* gene in pGK416-*ymUkG1* (via NsiI/NheI sites)	This study
pGK416m-p_{luxO10}-*ymUkG1*	p_{luxO10} was cloned upstream of *ymUkG1* gene in pGK416-*ymUkG1* (via NsiI and NheI sites)	This study
pATP416-*crtYBI*	pATP416 vector with $crtYB_{Xd}$, $crtI_{Xd}$, and *bts1* cloned downstream of p_{ADH1}, p_{TDH3}, and p_{PGK1}, respectively (see **Supplementary Fig. 21**)	This study
pATP416-p_{luxO5}-p_{phlO6}-*crtYBI*	pATP416 vector with $crtYB_{Xd}$, $crtI_{Xd}$, and *bts1* cloned downstream of p_{luxO5}, p_{TDH3}, and p_{phlO6}, respectively (see **Supplementary Fig. 21**)	This study
pATP416-p_{tetO7}-p_{phlO6}-*crtYBI*	pATP416 vector with $crtYB_{Xd}$, $crtI_{Xd}$, and *bts1* cloned downstream of p_{tetO7}, p_{TDH3}, and p_{phlO6}, respectively (see **Supplementary Fig. 21**)	This study
pATP416-p_{luxO5}-p_{tetO7}-*crtYBI*	pATP416 vector with $crtYB_{Xd}$, $crtI_{Xd}$, and *bts1* cloned downstream of p_{tetO7}, p_{TDH3}, and p_{phlO6}, respectively (see **Supplementary Fig. 21**)	This study
pACYCDuet-*luxR*-*ymUkG1*	pACYCDuet-1 vector with *luxR* and p_{lux}-*ymUkG1* cassette cloned via NcoI/AflII and HpaI/MluI sites, respectively	This study
pACYCDuet-$luxR_{S116Y}$-*ymUkG1*	pACYCDuet-1 vector with $luxR_{\mathrm{S116Y}}$ and p_{lux}-*ymUkG1* cassette cloned via NcoI/AflII and HpaI/MluI sites, respectively	This study
pACYCDuet-$luxR_{S116Y,\ W201R}$-*ymUkG1*	pACYCDuet-1 vector with $luxR_{\mathrm{S116Y,\ W201R}}$ and p_{lux}-*ymUkG1* cassette cloned via NcoI/AflII and HpaI/MluI sites, respectively.	This study

Supplementary Table 4. DNA sequences used in this study.

Name	Sequence	Notes
TBG gene	ctgcagatggcttcttatccaggtcatcaacatgcttcagcttttgatca<br>agctgctagatcaagaggtcattctaatagaagaaccgccttaagaccaa<br>gaagacaacaagaagctactgaagttagaccagaacaaaagatgccaact<br>ttgttgagagtttacattgatggtccacatggtatgggtaagactactac<br>tactcaattattggttgccttgggttccagagatgatatcgtttatgttc<br>cagaacctatgacctattggagagttttgggtgcttctgaaactattgct<br>aacatctacactacccaacacagattggatcaaggtgaaatttctgctgg<br>tgatgctgctgttgttatgacttctgctcaaattactatgggtatgccat<br>acgctgttactgatgctgttttggctccacatattggtggtgaagctggt<br>tcttctcatgctccaccaccagctttgactttgattttgatagacatcc<br>aattgccgccttgttgtgttatccagcagctagatatttgatgggttcta<br>tgactccacaagccgttttggcttttgttgctttgattccaccaactttg<br>ccaggtactaatatcgttttaggtgctttgccagaagatagacatattga<br>tagattggccaagagacaaagaccaggtgaaagattggatttggctatgt<br>tggctgctatcagaagagtttacggtttgttggctaacaccgttagatac<br>ttgcaatgtggtggttcttggagagaagattggggtcaattgtctggtac<br>tgctgttccaccacaaggtgctgaaccacaatctaatgctggtccaagac<br>cacatataggtgatactttgtttaccttgtttagagccccagaattattg<br>gctccaaatggtgacttgtacaatgtttttgcttgggccttggatgtttt<br>agccaaaagattgagatccatgcacgttttcatcttggattacgatcaat<br>ctccagcaggttgtagagatgctttgttgcaattgacttctggtatggtt<br>caaactcatgttactaccccaggttctattccaaccatttgtgatttggc<br>aagaaccttcgctagagaaatgggtgaagctaacgctaaattgacttccg<br>ctgttccagttttgactgctagagatgttgctggtgctgttgaattttgg<br>actgctagattgggtttctccagagatttcgttgaagatgatttcgctgg<br>tgttgttagagatgatgttaccttgttcatttccgccgttcaagatcaag<br>ttgttccagataatactttggcttgggtttgggttagaggtttggatgaa<br>ttatatgccgaatggtccgaagttgtttctaccaattttagagatgcttc<br>tggtccagctatgaccgaaattggtgaacaaccatggggtagagaatttg<br>ctttgagagatccagctggtaattgcgttcattttgttgctgaagaacaa<br>gatgtcagtgtcatcaaagaagaaatgaagatcaagttgcacatggaagg<br>taacgttaatggtcatgcctttgttattgaaggtgatggtaaaggtaaac<br>catacgatggtactcaaactttgaacttgactgtcaaagaaggtgctcca<br>ttgccattctcttacgatattttgactaacgccttccaatacggtaatag<br>agcttttactaagtacccagccgatatcccagattactttaagcaaactt<br>ttccagaaggttactcctgggaaagaactatgtcttacgaagataacgct<br>atctgcaacgtcagatccgaaatttctatggaaggtgattgcttcatcta<br>caagatcagattcgatggtaagaactttccaccaaatggtccagtcatgc	PstI, *hsvtk*, *ble*, *ymUkG1*, T_{CYC1}, EagI

	**aaaaaaagactttgaagtgggaaccatccaccgaaatgatgtatgttaga**<br>**gatggtttcttgatgggtgatgtcaatatggctttgttgttggaaggtgg**<br>**tggtcatcatagatgtgatttcaagacttcttacaaggccaagaaggttg**<br>**ttcaattgccagatgctcataagatcgatcacagaatcgaaatcttgtcc**<br>**cacgatagagattactccaaggttaagttgtacgaaaacgctgttgctag**<br>**aaactctttgttgccatctcaagcttctaagtaa**GTCGACCTCGAGTcat<br>gtaattagttatgtcacgcttacattcacgccctccccccacatccgctc<br>taaccgaaaaggaaggagttagacaacctgaagtctaggtccctatttat<br>ttttttatagttatgttagtattaagaacgttatttatatttcaaatttt<br>tctttttttctgtacagacgcgtgtacgcatgtaacattatactgaaaa<br>ccttgcttgagaaggttttgggacgctcgaaggctttaatttgcggccg	
hENT1 expression cassette	gaataaaaaacacgcttttcagttcgagtttatcattatcaatactgcc<br>atttcaaagaatacgtaaataattaatagtagtgattttcctaactttat<br>ttagtcaaaaaattagccttttaattctgctgtaacccgtacatgcccaa<br>aataggggggcgggttacacagaatatataacatcgtaggtgtctgggtga<br>acagtttattcctggcatccactaaatataatggagcccgctttttaagc<br>tggcatccagaaaaaaaaagaatcccagcaccaaaatattgttttcttca<br>ccaaccatcagttcataggtccattctcttagcgcaactacagagaacag<br>gggcacaaacaggcaaaaaacgggcacaacctcaatggagtgatgcaacc<br>tgcctggagtaaatgatgacacaaggcaattgacccacgcatgtatctat<br>ctcattttcttacaccttctattaccttctgctctctctgatttggaaaa<br>agctgaaaaaaaaggttgaaaccagttccctgaaattattcccctacttg<br>actaataagtatataaagacggtaggtattgattgtaattctgtaaatct<br>atttcttaaacttcttaaattctacttttatagttagtcttttttttagt<br>tttaaaacaccaagaacttagtttcgaataaacacacataAACAAACAAA<br>GTCGA**atgacaaccagtcaccagcctcaggacagatacaaagctgtctgg**<br>**cttatcttcttcatgctgggtctgggaacgctgctcccgtggaatttttt**<br>**catgacggccactcagtatttcacaaaccgcctggacatgtcccagaatg**<br>**tgtccttggtcactgctgaactgagcaaggacgcccaggcgtcagccgcc**<br>**cctgcagcaccccttgcctgagcggaactctctcagtgccatcttcaGcaa**<br>**tgtcGtgaccctatgtgccatgctgcccctgctgttattcacctacctca**<br>**actccttcctgcatcagaggatcccccagtccgtacggatcctgggcagc**<br>**ctggtggccatcctgctggtgtttctgatcactgccatcctggtgaaggt**<br>**gcagctggatgctctgcccttctttgtcatcaccatgatcaagatcgtgc**<br>**tcattaattcatttggtgccatcctgcagggcagcctgtttggtctggct**<br>**ggccttctgcctgccagctacacggcccccatcatgagtggccagggcct**<br>**agcaggcttctttgcctccgtggccatgatctgcgctattgccagtggct**<br>**cggaGctatcagaaagtgccttcggctactttatcacagcctgtgctgtt**<br>**atcattttgaccatcatctgttacctgggcctgccccgcctggaattcta**<br>**ccgctactaccagcagctcaagcttgaaggacccggggagcaggagacca**	p_{TDH3}, *hENT1*, MluI, NotI, T_{TDH3},

	**agtCggacctcattagcaaaggagaggagccaagagcaggcaaagaggaatctggagtttcagtctccaactctcagcccaccaatgaaagccactctatcaaagccatcctgaaaaatatctcagtcctggctttctctgtctgcttcatcttcactatcaccattgggatgtttccagccgtgactgttgaggtcaagtccagcatcgcaggcagcagcacctgggaacgttacttcattcctgtgtcctgtttcttgactttcaatatctttgactggttgggccggagcctcacagctgtattcatgtggcctgggaaggacagccgctggctgccaagcctggtgctggcccggctggtgtttgtgccactgctgctgctgtgcaacattaagccccgccgctacctgactgtggtcttcgagcacgatgcctggttcatcttcttcatggctgcctttgccttctccaacggctacctcgccagcctctgcatgtgcttcgggcccaagaaagtgaagccagctgaggcagGgaccgcaggagccatcatggccttcttcctgtgtctgggtctggcactgggggctgttttctccttcctgttccgggcaattgtgtaa**TCGACacgcgtgcggccgcgtgaatttactttaaatcttgcatttaaataaatttctttttatagctttatgacttagtttcaatttatatactattttaatgacattttcgattcattgattgaaagctttgtgtttttttcttgatgcgctattgcattgttcttgtcttttttcgccacatgtaatatctgtagtagatacctgatacattgtggatgctgagtgaaattttagttaataatggaggcgctcttaataattttggggatattggcttttttttttaaagtttacaaatgaatttttttccgccaggat	
p_{tetO7}	**ccattcgccattcaggctgcgcaactgttgggaagggcgatcggtgcgggcctcttcgctattacgcca**GCGGATCCCGACTCACTATAGGGCGAATTGggtaccGGGCCCCCCCTCGAGTTTAC*tccctatcagtgatagaga*aCGTATGTCGAGTTTAC*tccctatcagtgatagagaa*CGATGTCGAGTTTAC*tccctatcagtgatagaga*aCGTATGTCGAGTTTAC*tccctatcagtgatagaga*aCGTATGTCGAGTTTAC*tccctatcagtgatagaga*aCGTATGTCGAGTTTA*tccctatcagtgatagaga*aCGTATGTCGAGTTTAC*tccctatcagtgatagagaa*CGTATGTggatgataatgcgattagtttttttagccttatttctggggtaattaatcagcgaagcgatgatttttgatctattaacagatatataaatgcaaaaactgcataaccactttaActaatactttcaacattttcggtttgtattacttcttattcaaatgtaataaaagtatcaacaaaaaattgttaatatacctctatactttaacgtcaaggagaaaaaactatactgcag	*lacZα*, BamHI, *tetO*, p_{GAL1-c}, PstI
p_{camO1}	**ccattcgccattcaggctgcgcaactgttgggaagggcgatcggtgcgggcctcttcgctattacgcca**GCggatcc*caggctctatatctgcgatatactgagcat*ggatgataatgcgattagtttttttagccttatttctggggtaattaatcagcgaagcgatgatttttgatctattaacagatatataaatgcaaaaactgcataaccactttaactaatactttcaacattttcggtttgtattacttcttattcaaatgtaataaaagtatcaacaaaaaattgttaatatacctctatactttaacgtcaaggagaaaaaactatactgcag	*lacZα*, BamHI, *camO1*, p_{GAL1-c}, PstI
p_{phlO1}	**ccattcgccattcaggctgcgcaactgttgggaagggcgatcggtgcggg**	*lacZα*, BamHI,

	**cctcttcgctattacgcca**GCggatccTATGT*atgatacgaaacgtaccgtatcgttaaggt*AGCGTggatgataatgcgattagtttttagccttatttctggggtaattaatcagcgaagcgatgattttgatctattaacagatatataaatgcaaaaactgcataaccactttaactaatactttcaacattttcggtttgtattacttcttattcaaatgtaataaaagtatcaacaaaaaattgttaatatacctctatactttaacgtcaaggagaaaaaactatactgcag	*phlO1*, $p_{GAL1\text{-}c}$, PstI
p_{luxO1}	**ccattcgccattcaggctgcgcaactgttgggaagggcgatcggtgcgggcctcttcgctattacgcca**GCggatcc*acctgtaggatcgtacaggt*ggatgataatgcgattagtttttagccttatttctggggtaattaatcagcgaagcgatgattttgatctattaacagatatataaatgcaaaaactgcataaccactttaactaatactttcaacattttcggtttgtattacttcttattcaaatgtaataaaagtatcaacaaaaaattgttaatatacctctatactttaacgtcaaggagaaaaaactatactgcag	*lacZα*, BamHI, *luxO1*, $p_{GAL1\text{-}c}$, PstI
p_{PGK1}	aaagatgccgatttgggcgcgaatcctttattttggcttcaccctcatactattatcagggccagaaaaaggaagtgtttccctccttcttgaattgatgttaccctcataaagcacgtggcctcttatcgagaaagaaattaccgtcgctcgtgatttgtttgcaaaaagaacaaaactgaaaaaacccagacacgctcgacttcctgtcttcctattgattgcagcttccaatttcgtcacacaacaaggtcctagcgacggctcacaggttttgtaacaagcaatcgaaggttctggaatggcgggaaagggtttagtaccacatgctatgatgcccactgtgatctccagagcaaagttcgttcgatcgtactgttactctctctctttcaaacagaattgtccgaatcgtgtgacaacaacagcctgttctcacacactcttttcttctaaccaagggggtggtttagtttagtagaacctcgtgaaacttacatttacatatatataaacttgcataaattggtcaatgcaagaaatacatatttggtcttttctaattcgtagtttttcaagttcttagatgctttctttttctctttttacagatcatcaaggaagtaattatctacttttttacaacaaatataAAACgctagcgtcgac	p_{PGK1}, NheI, SalI
T_{PGK1}	agatctgaaataaattgaattgaattgaaatcgatagatcaatttttttcttttctctttccccatcctttacgctaaaataatagtttattttattttttgaatatttttatttatatacgtatatatagactattatttatcttttaatgattattaagatttttattaaaaaaaaattcgctcctcttttaatgcctttatgcagtttttttttcccattcgatatttctatgttcgggttcagcgtattttaagtttaataactcgaaaattctgcgttcgttaaagct	BglII, T_{PGK1}

rTetTA (*rtTA2^S-M2*)	gtcgac**atgtctagactggacaagagcaaagtcataaacggcgctctggaattactcaatggagtcggtatcgaaggcctgacgacaaggaaactcgctcaaaagctgggagttgagcagcctaccctgtactggcacgtgaagaacaagcgggccctgctcgatgccctgccaatcgagatgctggacaggcatcataccccacttctgccccctggaaggcgagtcatggcaagactttctgcggaacaacgccaagtcattccgctgtgctctcctctcacatcgcgacggggctaaagtgcatctcggcacccgcccaacagagaaacagtacgaaaccctggaaaatcagctcgcgttcctgtgtcagcaaggcttctccctggagaacgcactgtacgctctgtccgccgtgggccactttacactgggctgcgtattggaggaacaggagcatcaagtagcaaaagaggaaagagagacacctaccaccgattctatgcccccacttctgagacaagcaattgagctgttcgaccggcagggagccgaacctgccttccttttcggcctggaactaatcatatgtggcctggagaaacagctaaagtgcgaaagcggcgggccggccgacgcccttgacgattttgacttagacatgctcccagccgatgcccttgacgactttgaccttgatatgctgcctgctgacgctcttgacgattttgaccttgacatgctccccgggtaa**CTAAGTAagatct	SalI, *rtetR*, 3× VP16, BglII
phlTA	gtcgac**atggctagaactccatccagatcatctattggttctttgagatcaccacatactcataaggctattttgacctccaccatcgaaatcttgaaagaatgtggttactccggtttgtccattgaatctgttgctagacgtgctggtgcttctaaacctactatctacagatggtggactaacaaggctgctttgattgctgaagtttacgaaaacgaatccgaacaggttagaaagtttccagatttgggttcctttaaggccgatttggatttcttgttgagaaacttgtggaaggtttggagagaaactatttgtggtgaagccttcagatgcgttattgcagaagctcaattagatccagctactttgactcaattgaaggaccaattcatggaaagacgtagagaaatgccaaaaaagttggttgaaaacgccatctctaatggtgaattgccaaaggatacaaaccgtgagttgttgttggatatgattttcggtttttgctggtacaggttgttgactgaacaattgactgttgaacaagacatcgaagagttcacgttcttgttaatcaatggtgtttgtccaggtactcaaagaggtccagcagacgctttggatgattttgatttggatatgttgcctgctgatgccttggatgacttcgacttagacatgttaccagccgatgcattagacgatttcgaccttgatatgttacctggtccaccaaaaaagaagagaaaggtctaa**gatct	SalI, *phlF*, 3× VP16, NLS, BglII
camTA	gtcgac**atggacatcaagcaatctttgttgcatgctgccatgagattattgtctgctaaaggtagagatggtgctactatgaggccaatttgtgctgaagttggtgttactccaccaaccttgtatcatcattatggtgacttgcaaggtttacacaaagctgctattgacgaaacctatagacaagttgctgaagcctatcatggtggtactgaagaaagaggtccattgaaaggtataagagatggttgggctactttcttgcaatttgcttactctgaacctaacatgtgcagaatgttggttcaacatattatggctggtgaaccaccatctatggttgctgatactttgagaggtgttgctgatgatttggctcaatttcatgctcaaggtagat**	SalI, *camR*, 3× VP16, NLS, BglII

	tgacttttccaccaagagaagctgctcaattattgtggatgggtgcttta<br>ggtgctttgacttatgctttgtcaagagaaggtgcaggttacactcaaga<br>tttggctttacaaaaggccaagttggatattaccttggttgccttgttca<br>acattgaagaagaaggtccagcagacgctttggatgattttgatttggat<br>atgttgcctgctgatgccttggatgacttcgacttagacatgttaccagc<br>cgatgcattagacgattttgaccttgacatgttgccaggtccaccaaaaa<br>agaaaagaaaggtctaagatct	
camTA (-nls)	gtcgacatggacatcaagcaatctttgttgcatgctgccatgagattatt<br>gtctgctaaaggtagagatggtgctactatgaggccaatttgtgctgaag<br>ttggtgttactccaccaaccttgtatcatcattatggtgacttgcaaggt<br>ttacacaaagctgctattgacgaaacctatagacaagttgctgaagccta<br>tcatggtggtactgaagaaagaggtccattgaaaggtataagagatggtt<br>gggctactttcttgcaatttgcttactctgaacctaacatgtgcagaatg<br>ttggttcaacatattatggctggtgaaccaccatctatggttgctgatac<br>tttgagaggtgttgctgatgatttggctcaatttcatgctcaaggtagat<br>tgacttttccaccaagagaagctgctcaattattgtggatgggtgcttta<br>ggtgctttgacttatgctttgtcaagagaaggtgcaggttacactcaaga<br>tttggctttacaaaaggccaagttggatattaccttggttgccttgttca<br>acattgaagaagaaggtccagcagacgctttggatgattttgatttggat<br>atgttgcctgctgatgccttggatgacttcgacttagacatgttaccagc<br>cgatgcattagacgattttgaccttgacatgttgccaggtccaccaaaaa<br>agaaaagaaaggtctaagatct	SalI, *camR*, 3× VP16, BglII
luxTA	gtcgacatgccagccgatgctttggatgatttcgatttggatatgttgcc<br>tgctgatgcattggacgattttgacttagacatgttaccagcagacgcat<br>tggatgacttcgaccttgatatgctaccaggtccaccaaaaaagaagaga<br>aaggttgaaaacatcaacgctgatgacacctacagaatcatcaacaagat<br>taaggcttgcaggtctaacaacgatattaaccagtgtttgtccgacatga<br>ccaagatggttcattgcgaatattacttgttggccatcatctacccacac<br>tctatggttaagtccgacatttccattttggacaactacccaaaaaaatg<br>gcgtcagtattacgatgatgccaacttgattaagtacgacccaatcgttg<br>attactccaactctaatcattctcccatcaactggaacatcttcgaaaac<br>aatgccgtcaacaaaaagtccccaaacgttatcaaagaagctaagacctc<br>tggtttgattaccggtttttctttcccaattcataccgctaacaatggtt<br>tcggtatgttgtcttttgcccactctgaaaaggataactacatcgactca<br>ttattcttgcatgcctgcatgaacattccattgatagttccatctctggt<br>cgataactataggaagattaacattgccaacaacaagtccaacaacgatt<br>tgaccaaaagggaaaaagaatgtttggcttgggcttgcgaaggtaaatct<br>tcttgggatatttccaagattttgggttgctctgaaagaaccgttacttt<br>ccatttgactaacgcccaaatgaagttgaacactaccaacagatgccagt<br>ctatttctaaggctattttgaccggtgctattgattgtccatactttaag	SalI, 3× VP16, NLS, *luxR*, BglII

	**aatta**agatct	
p_{tetO7}-*ymUkG1*	aaaactgtattataagtaaaCTATTACGCCAGCGGATCCCGACTCACTATAGGGCGAATTGGGTACCGGGCCCCCCCTCGAGTTTAC*tccctatcagtgatagagaa*CGTATGTCGAGTTTAC*tccctatcagtgatagagaa*CGATGTCGAGTTTAC*tccctatcagtgatagagaa*CGTATGTCGAGTTTAC*tccctatcagtgatagagaa*CGTATGTCGAGTTTAC*tccctatcagtgatagagaa*CGTATGTCGAGTTTA*tccctatcagtgatagagaa*CGTATGTCGAGTTTAC*tccctatcagtgatagagaa*CGTATGTggatgataatgcgattagttttttagccttatttctggggtaattaatcagcgaagcgatgatttttgatctattaacagatatataaatgcaaaaactgcataaccactttaactaatactttcaacattttcggtttgtattacttcttattcaaatgtaataaaagtatcaacaaaaaattgttaatatacctctatactttaacgtcaaggagaaaaaactatagctagcgtcgac**atg**	*URA3* marker, *tetO*, p_{GAL1-c}, NheI, SalI, start codon of *ymUkG1*
p_{phlO6}-*ymUkG1*	aaaactgtattataagtaaaCTATTACGCCAGCGGATCC*atgatacgaaacgtaccgtatcgttaaggt*GA*atgatacgaaacgtaccgtatcgttaaggt*CA*atgatacgaaacgtaccgtatcgttaaggt*CA*atgatacgaaacgtaccgtatcgttaaggt*GT*atgatacgaaacgtaccgtatcgttaaggt*AGa*tgatacgaaacgtaccgtatcgttaaggt*ggcgttgtgatccggatgataatgcgattagttttttagccttatttctggggtaattaatcagcgaagcgatgatttttgatctattaacagatatataaatgcaaaaactgcataaccactttaactaatactttcaacattttcggtttgtattacttcttattcaaatgtaataaaagtatcaacaaaaaattgttaatatacctctatactttaacgtcaaggagaaaaaactatagctagcgtcgac**atg**	*URA3* marker, *phlO6*, p_{GAL1-c}, NheI, SalI, start codon of *ymUkG1*
P_{luxO5}-*ymUkG1*	aaaactgtattataagtaaaCTGAAAAAGCGTGTTTTTTATTCCTTAAGTTACGCAAGGTCGAACATAAGT*acctgtaggatcgtacaggt*TTACGCAAGGTCGAACATAAGT*acctgtaggatcgtacaggt*CGCTATTACGCCAGCGGATCC*acctgtaggatcgtacaggt*GA*acctgtaggatcgtacaggt*CA*acctgtaggatcgtacaggt*ACGTATGTGATCCggatgataatgcgattagttttttagccttatttctggggtaattaatcagcgaagcgatgatttttgatctattaacagatatataaatgcaaaaactgcataaccactttaactaatactttcaacattttcggtttgtattacttcttattcaaatgtaataaaagtatcaacaaaaaattgttaatatacctctatactttaacgtcaaggagaaaaaactatagctagcgtcgac**atg**	*URA3* marker, *luxO5*, p_{GAL1-c}, NheI, SalI, start codon of *ymUkG1*
P_{luxO10}-*ymUkG1*	aaaactgtattataagtaaaCTGAAAAAGCGTGTTTTTTATTCCTTAAGTTACGCAAGGTCGAACATAAGT*acctgtaggatcgtacaggt*TTACGCAAGGTCGAACATAAGT*acctgtaggatcgtacaggt*cgctattacgccagcggatCc*acctgtaggatcgtacaggt*GA*acctgtaggatcgtacaggt*CA*acctgtaggatcgtacaggt*ACGTATGTGATCCggatgataatgttaagTTACGCAAGGTCGAACATAAGT*acctgtaggatcgtacaggt*TTACGCAAGGT	*URA3* marker, *luxO10*, p_{GAL1-c}, NheI, SalI, start codon of *ymUkG1*

	CGAACATAAGT*acctgtaggatcgtacaggt*cgctattacgccagcggat<br>Cc*acctgtaggatcgtacaggt*GA*acctgtaggatcgtacaggt*CA*acct*<br>*gtaggatcgtacaggt*ACGTATGTGATCCggatgataatgcgattagttt<br>tttagccttatttctggggtaattaatcagcgaagcgatgatttttgatc<br>tattaacagatatataaatgcaaaaactgcataaccactttaactaatac<br>tttcaacattttcggtttgtattacttcttattcaaatgtaataaaagta<br>tcaacaaaaaattgttaatatacctctatactttaacgtcaaggagaaaa<br>aactatagctagcgtcgac**atg**	
crtYB	cctagg**atgacggctctcgcatattaccagatccatctgatctatactct**<br>**cccaattcttggtcttctcggtctgctcacttccccgattttgacaaaat**<br>**ttgacatctacaaaatatcgatcctcgtatttattgcgtttagtgcaacc**<br>**acaccatgggactcatggatcatcagaaatggcgcatggacatatccatc**<br>**agcggagagtggccaaggcgtgtttggaacgtttctagatgttccatatg**<br>**aagagtacgctttctttgtcattcaaaccgtaatcaccggcttggtctac**<br>**gtcttggcaactaggcaccttctcccatctctcgcgcttcccaagactag**<br>**atcgtccgccctttctctcgcgctcaaggcgctcatccctctgcccatta**<br>**tctacctatttaccgctcaccccagcccatcgcccgacccgctcgtgaca**<br>**gatcactacttctacatgcgggcactctccttactcatcaccccacctac**<br>**catgctcttggcagcattatcaggcgaatatgctttcgattggaaaagtg**<br>**gccgagcaaagtcaactattgcagcaatcatgatcccgacggtgtatctg**<br>**atttgggtagattatgttgctgtcggtcaagactcttggtcgatcaacga**<br>**tgagaagattgtagggtggaggcttggaggtgtactacccattgaggaag**<br>**ctatgttcttcttactgacgaatctaatgattgttctgggtctgtctgcc**<br>**tgcgatcatactcaggccctatacctgctacacggtcgaactatttatgg**<br>**caacaaaaagatgccatcttcatttcccctcattacaccgcctgtgctct**<br>**ccctgtttttagcagccgaccatactcttctcagccaaaacgtgacttg**<br>**gaactggcagtcaagttgttggaggaaaagagccggagctttttgttgc**<br>**ctcggctggatttcctagcgaagttagggagaggctggttggactatacg**<br>**cattctgccgggtgactgatgatcttatcgactctcctgaagtatcttcc**<br>**aacccgcatgccacaattgacatggtctccgattttcttaccctactatt**<br>**tgggcccccgctacacccttcgcaacctgacaagatcctttcttcgcctt**<br>**tacttcctccttcgcacccttcccgacccacgggaatgtatcccctcccg**<br>**cctcctccttcgctctcgcctgccgagctcgttcaattccttaccgaaag**<br>**ggttcccgttcaataccatttcgccttcaggttgctcgctaagttgcaag**<br>**ggctgatccctcgatacccactcgacgaactccttagaggatacaccact**<br>**gatcttatctttcctttatcgacagaggcagtccaggctcggaagacgcc**<br>**tatcgagaccacagctgacttgctggactatggtctatgtgtagcaggct**<br>**cagtcgccgagctattggtctatgtctcttgggcaagtgcaccaagtcag**<br>**gtccctgccaccatagaagaaagagaagctgtgttagtggcaagccgaga**<br>**gatgggaactgcccttcagttggtgaacattgctagggacattaaaggg**	AvrII, *crtYB*, FseI

	**acgcaacagaagggagattttacctaccactctcattctttggtcttcgg**<br>**gatgaatcaaagcttgcgatcccgactgattggacggaacctcggcctca**<br>**agatttcgacaaactcctcagtctatctccttcgtccacattaccatctt**<br>**caaacgcctcagaaagcttccggttcgaatggaagacgtactcgcttcca**<br>**ttagtcgcctacgcagaggatcttgccaaacattcttataagggaattga**<br>**ccgacttcctaccgaggttcaagcgggaatgcgagcggcttgcgcgagct**<br>**acctactgatcggccgagagatcaaagtcgtttggaaaggagacgtcgga**<br>**gagagaaggacagttgccggatggaggagagtacggaaagtcttgagtgt**<br>**ggtcatgagcggatgggaagggcagtaa**ggccggcc	
crtI	acgcgt**atgggaaaagaacaagatcaggataaacccacagctatcatcgt**<br>**gggatgtggtatcggtggaatcgccactgccgctcgtcttgctaaagaag**<br>**gtttccaggtcacggtgttcgagaagaacgactactccggaggtcgatgc**<br>**tctttaatcgagcgagatggttatcgattcgatcaggggcccagtttgct**<br>**gctcttgccagatctcttcaagcagacattcgaagatttgggagagaaga**<br>**tggaagattgggtcgatctcatcaagtgtgaacccaactatgtttgccac**<br>**ttccacgatgaagagactttcactctttcaaccgacatggcgttgctcaa**<br>**gcgggaagtcgagcgttttgaaggcaaagatggatttgatcggttcttgt**<br>**cgtttatccaagaagcccacagacattacgagcttgctgtcgttcacgtc**<br>**ctgcagaagaacttccctggcttcgcagcattcttacggctacagttcat**<br>**tggccaaatcctggctcttcaccccttcgagtctatctggacaagagttt**<br>**gtcgatatttcaagaccgacagattacgaagagtcttctcgtttgcagtg**<br>**atgtacatgggtcaaagcccatacagtgcgcccggaacatattccttgct**<br>**ccaatacaccgaattgaccgagggcatctggtatccgagaggaggctttt**<br>**ggcaggttcctaatactcttcttcagatcgtcaagcgcaacaatccctca**<br>**gccaagttcaatttcaacgctccagtttcccaggttcttctctctcctgc**<br>**caaggaccgagcgactggtgttcgacttgaatccggcgaggaacatcacg**<br>**ccgatgttgtgattgtcaatgctgacctcgtttacgcctccgagcacttg**<br>**attcctgacgatgccagaaacaagattggccaactgggtgaagtcaagag**<br>**aagttggtgggctgacttagttggtggaaagaagctcaagggaagttgca**<br>**gtagtttgagcttctactggagcatggaccgaatcgtggacggtctgggc**<br>**ggacacaatatcttcttggccgaggacttcaagggatcattcgacacaat**<br>**cttcgaggagttgggtctcccagccgatccttccttttacgtgaacgttc**<br>**cctcgcgaatcgatccttctgccgctcccgaaggcaaagatgctatcgtc**<br>**attcttgtgccgtgtggccatatcgacgcttcgaaccctcaagattacaa**<br>**caagcttgttgctcgggcaaggaagtttgtgatccaaacgctttccgcca**<br>**agcttggacttcccgactttgaaaaaatgattgtggcagagaaggttcac**<br>**gatgctccctcttgggagaaagaattcaacctcaaggacggaagcatctt**<br>**gggactggctcacaactttatgcaagttcttggtttcaggccgagcacca**<br>**gacatcccaagtatgacaagttgttctttgtcggggcttcgactcatccc**<br>**ggaactggggttcccatcgtcttggctggagccaagttaactgccaacca**	MluI, *crtI*, NotI

	**agttctcgaatcctttgaccgatccccagctccagatcccaatatgtcac**<br>**tctccgtaccatatggaaaacctctcaaatcaaatggaacgggtatcgat**<br>**tctcaggtccagctgaagttcatggatttggagagatgggtataccttttt**<br>**ggtattgttgattggggccgtgatcgctcgatccgttggtgttcttgctt**<br>**tctga**gcggccgc	
BTS1	gctagc**atggaggccaagatagatgagctgatcaataatgatcctgtttg**<br>**gtccagccaaaatgaaagcttgatttcaaaccttataatcacatccttt**<br>**tgaaacctggcaagaactttagactaaatttaatagttcaaattaacaga**<br>**gttatgaatttgcccaaagaccagctggccatagtttcgcaaattgttga**<br>**gctcttgcataattccagccttttaatcgacgatatagaagataatgctc**<br>**ccttgagaaggggacagaccacttctcacttaatcttcggtgtaccctcc**<br>**actataaacaccgcaaattatatgtatttcagagccatgcaacttgtatc**<br>**gcagctaaccacaaaagagcctttgtatcataatttgattacgattttca**<br>**acgaagaattgatcaatctacataggggacaaggcttggatatatactgg**<br>**agagactttctgcctgaaatcatacctactcaggagatgtatttgaatat**<br>**ggttatgaataaaacaggcggccttttcagattaacgttgagactcatgg**<br>**aagcgctgtctccttcctcacaccacggccattcgttggttcctttcata**<br>**aatcttctgggtattatttatcagattagagatgattacttgaatttgaa**<br>**agatttccaaatgtccagcgaaaaaggctttgctgaggacattacagagg**<br>**ggaagttatcttttcccatcgtccacgcccttaacttcactaaaacgaaa**<br>**ggtcaaactgagcaacacaatgaaattctaagaattctcctgttgaggac**<br>**aagtgataaagatataaaactaaagctgattcaaatactggaattcgaca**<br>**ccaattcattggcctacaccaaaaatttattaatcaattagtgaatatg**<br>**ataaaaaatgataatgaaaataagtatttacctgatttggcttcgcattc**<br>**cgacaccgccaccaatttacatgacgaattgttatatataatagaccact**<br>**tatccgaattgtga**cccgggggcgcgcc	NheI, *BTS1*, SmaI, AscI
p_{TDH3}-p_{luxO5}-*crtYB*	tttgtttgtttatgtgtgtttattcgaaactaaGttcttggtgttttaaa<br>actaaaaaaaagactaactataaaagtagaatttaagaagtttaagaaat<br>agatttacagaattacaatcaatacctaccgtctttatatacttattagt<br>caagtaggggaataatttcagggaactggtttcaaccttttttttcagct<br>ttttccaaatcagagagagcagaaggtaatagaaggtgtaagaaaatgag<br>atagatacatgcgtgggtcaattgccttgtgtcatcatttactccaggca<br>ggttgcatcactccattgaggttgtgcccgtttttttgcctgtttgtgccc<br>ctgttctctgtagttgcgctaagagaatggacctatgaactgatggttgg<br>tgaagaaaacaatattttggtgctgggattcttttttttttctggatgcca<br>gcttaaaaagcgggctccattatatttagtggatgccaggaataaactgt<br>tcacccagacacctacgatgttatatattctgtgtaacccgcccccctatt<br>ttgggcatgtacgggttacagcagaattaaaaggctaatttttttgactaa<br>ataaagttaggaaaatcactactattaattatttacgtattctttgaaat<br>ggcagtattgataatgataaaCtcgaactgaaaaagcgtgtttttttattc	p_{TDH3}, AflII, *luxO*, p_{GAL1}, AvrII, start codon of *crtYB*

	cttaagTTACGCAAGGTCGAACATAAGT*acctgtaggatcgtacaggt*TT<br>ACGCAAGGTCGAACATAAGT*acctgtaggatcgtacaggt*CGCTATTACG<br>CCAGCGGATCC*acctgtaggatcgtacaggtgaacctgtaggatcgtaca*<br>*ggtcaacctgtaggatcgtacaggt*ACGTATGTGATCCggatgataatgc<br>gattagtttttttagccttatttctggggtaattaatcagcgaagcgatga<br>tttttgatctattaacagatatataaatgcaaaaactgcataaccacttt<br>aactaatactttcaacattttcggtttgtattacttcttattcaaatgta<br>ataaaagtatcaacaaaaaattgttaatatacctctatactttaacgtca<br>aggagaaaaaactatacctagg**atg**	
T_{PGK1}-p_{phlO6}-*BTS1*	gtgaatttactttaaatcttgcatttaaataaattttctttttatagctt<br>tatgacttagtttcaatttatatactattttaatgacattttcgattcat<br>tgattgaaagctttgtgtttttttcttgatgcgctattgcattgttcttgt<br>ctttttcgccacatgtaatatctgtagtagatacctgatacattgtggat<br>gctgagtgaaattttagttaataatggaggcgctcttaataattttgggg<br>atattggcttttttttttaaagtttacaaatgaattttttccgccaggat<br>ttaattaaCGCTATTACGCCAGCGGATCC*atgatacgaaacgtaccgtat*<br>*cgttaaggt*GA*atgatacgaaacgtaccgtatcgttaaggt*CA*atgatac*<br>*gaaacgtaccgtatcgttaaggt*CA*atgatacgaaacgtaccgtatcgtt*<br>*aaggt*GT*atgatacgaaacgtaccgtatcgttaaggt*AG*atgatacgaaa*<br>*cgtaccgtatcgttaaggt*GGCGTTGTGATCCggatgataatgcgattag<br>tttttttagccttatttctggggtaattaatcagcgaagcgatgatttttg<br>atctattaacagatatataaatgcaaaaactgcataaccactttaactaa<br>tactttcaacattttcggtttgtattacttcttattcaaatgtaataaaa<br>gtatcaacaaaaaattgttaatatacctctatactttaacgtcaaggaga<br>aaaaactatagctagc**atg**	T_{PGK1}, PacI, *phlO*, p_{GAL1-c}, NheI, start codon of *bts1*
T_{PGK1}-p_{tetO7}-*BTS1*	gtgaatttactttaaatcttgcatttaaataaattttctttttatagctt<br>tatgacttagtttcaatttatatactattttaatgacattttcgattcat<br>tgattgaaagctttgtgtttttttcttgatgcgctattgcattgttcttgt<br>ctttttcgccacatgtaatatctgtagtagatacctgatacattgtggat<br>gctgagtgaaattttagttaataatggaggcgctcttaataattttgggg<br>atattggcttttttttttaaagtttacaaatgaattttttccgccaggat<br>ttaattaaCGCTATTACGCCAGCGGATCCCGACTCACTATAGGGCGAATT<br>GGGTACCGGGCCCCCCCTCGAGTTTAC*tccctatcagtgatagaga*aCGT<br>ATGTCGAGTTTAC*tccctatcagtgatagagaa*CGATGTCGAGTTTAC*tc*<br>*cctatcagtgatagaga*aCGTATGTCGAGTTTAC*tccctatcagtgatag*<br>*aga*aCGTATGTCGAGTTTAC*tccctatcagtgatagaga*aCGTATGTCGA<br>GTTTA*tccctatcagtgatagaga*aCGTATGTCGAGTTTAC*tccctatca*<br>*gtgatagagaa*CGTATGTggatgataatgcgattagtttttttagccttat<br>ttctggggtaattaatcagcgaagcgatgatttttgatctattaacagat<br>atataaatgcaaaaactgcataaccactttaactaatactttcaacattt	T_{PGK1}, PacI, *tetO*, p_{GAL1-c}, NheI, start codon of *bts1*

	tcggtttgtattacttcttattcaaatgtaataaaagtatcaacaaaaaa<br>ttgttaatatacctctatactttaacgtcaaggagaaaaaactatagcta<br>gc**atg**	
p_{TDH3}-p_{tetO7}-*crtYB*	tttgtttgtttatgtgtgtttattcgaaactaagttcttggtgttttaaa<br>actaaaaaaaagactaactataaaagtagaatttaagaagtttaagaaat<br>agatttacagaattacaatcaatacctaccgtctttatatacttattagt<br>caagtaggggaataatttcagggaactggtttcaaccttttttttcagct<br>ttttccaaatcagagagagcagaaggtaatagaaggtgtaagaaaatgag<br>atagatacatgcgtgggtcaattgccttgtgtcatcatttactccaggca<br>ggttgcatcactccattgaggttgtgcccgtttttttgcctgtttgtgccc<br>ctgttctctgtagttgcgctaagagaatggacctatgaactgatggttgg<br>tgaagaaaacaatattttggtgctgggattcttttttttttctggatgcca<br>gcttaaaaagcgggctccattatatttagtggatgccaggaataaactgt<br>tcacccagacacctacgatgttatatattctgtgtaacccgcccccctatt<br>ttgggcatgtacgggttacagcagaattaaaaggctaattttttgactaa<br>ataaagttaggaaaatcactactattaattatttacgtattctttgaaat<br>ggcagtattgataatgataaaCtcgaactgaaaaagcgtgtttttattc<br>cttaagCGCTATTACGCCAGCGGATCCCGACTCACTATAGGGCGAATTGG<br>GTACCGGGCCCCCCCTCGAGtttac*tccctatcagtgatagaga*acgtat<br>gtcgagtttac*tccctatcagtgatagaga*acgatgtcgagtttac*tccc*<br>*tatcagtgatagaga*acgtatgtcgagtttac*tccctatcagtgatagag*<br>*a*acgtatgtcgagtttac*tccctatcagtgatagaga*acgtatgtcgagt<br>tta*tccctatcagtgatagaga*acgtatgtcgagtttac*tccctatcagt*<br>*gatagaga*ACGTATGTggatgataatgcgattagtttttttagccttattt<br>ctggggtaattaatcagcgaagcgatgatttttgatctattaacagatat<br>ataaatgcaaaaactgcataaccactttaactaatactttcaacattttc<br>ggtttgtattacttcttattcaaatgtaataaaagtatcaacaaaaaatt<br>gttaatatacctctatactttaacgtcaaggagaaaaaactatacctagg<br>**atg**	p_{TDH3}, AflII, *tetO*, p_{GAL1-c}, AvrII, start codon of *crtYB*
MET15red	cctcaaagttactgacgaagcttatgatggtttggttattgttattggta<br>gatggattcaagggtttttgtcccaaaagaataataacactattgaaggc<br>ttgaacttcatcagattacttgaaaaagattcaggtaaatctgagttctt<br>attaagctacggtaaggaagtaaacaaaattccacaaagctacgaaaatt<br>tgaagaaaggttccactgtaaccagcaatgggttgaactgggaagttatt<br>gaatatcacgcttaataaaggagaataaatcgttttctactttcttctgc<br>tgctataataagcacctatgggatctatatagtatttttataacgataga<br>ctttataaaagaaaatacctaagtgaaaatttggtgaattttgagataat<br>tgttgggattccatttttaataaggcaataatattaggtatgtagaatat<br>actagaagttctcctcgaggatttaggaatccataaaagggaatctgcaa<br>ttctacacaattctataaatattattatcatcgttttatatgttaatatt	

	cattgatcctattacattatcaatccttgcgtttcagcttccactaattt<br>agatgactatttctcatcatttgcgtcatcttctaacaccgtatatgata<br>atatactagtaacgtaaatactagttagtagatgatagttgatttttatt<br>ccaacactaagaaataatttcgccatttcttgaatgtatttaaagatatt<br>taatgctataatagacatttaaatccaattcttccaacatacaatgggag<br>tttggccgagtggtttaaggcgtcagatttaggtggatttaacctctaaa<br>atctctgatatcttcggatgcaagggttcgaatcccttagctctcattat<br>tttttgctttttctcttgaggtcacatgatcgcaaaatggcaaatggcac<br>gtgaagctgtcgatattggggaactgtggtggttggcaaatgactaatta<br>agttagtcaaggcgccatcctcatgaaaactgtgtaacataataaccgaa<br>gtgtcgaaaaggtggcaccttgtccaattgaacacgctcgatgaaaaaaa<br>taagatatatataaggttaagtaaagcgtctgttagaaaggaagtttttc<br>ctttttcttgctctcttgtcttttcatctactatttccttcgtgtaatac<br>agggtcgtcagatacatagatacaattctattacccccatccatacaa**atg**<br>**ccatctcatttcgatactgttcaactacacgccggccaagagaaccctgg**<br>**tgacaatgctcacagatccagagctgtaccaatttacgccaccacttctt**<br>**atgttttcgaaaactctaagcatggttcgcaattgtttggtctagaagtt**<br>**ccaggttacgtctattcccgtttccaaaacccaaccagtaatgttttgga**<br>**agaaagaattgctgctttagaaggtggtgctgctgctttggctgtttcct**<br>**ccggtcaagccgctcaaacccttgccatccaaggtttggcacacactggt**<br>**gacaacatcgtttccacttcttacttatacggtggtacttataaccagtt**<br>**caaaatctcgttcaaaagatttggtatcgaggctagatttgttgaaggtg**<br>**acaatccagaagaattcgaaaaggtctttgatgaaagaaccaaggctgtt**<br>**tatttggaaaccattggtaatccaaagtacaatgttccggattttgaaaa**<br>**aattgttgcaattgctcacaaacacggtattccagttgtcgttgacaaca**<br>**catttggtgccggtggttacttctgtcagccaattaaatacggtgctgat**<br>**attgtaacacattctgctaccaaatggattggtggtcatggtactactat**<br>**cggtggtattattgttgactctggtaagttcccatggaaggactacccag**<br>**aaaagttccctcaattctctcaacctgccgaaggatatcacggtactatc**<br>**tacaatgaagcctacggtaacttggcatacatcgttcatgttagaactga**<br>**actattaagagatttgggtccattgatgaacccatttgcctctttcttgc**<br>**tactacaaggtgttgaaacattatctttgagagctgaaagacacggtgaa**<br>**aatgcattgaagttagccaaatggttagaacaatccccatacgtatcttg**<br>**ggtttcataccctggtttagcatctcattctcatcatgaaaatgctaaga**<br>**agtatctatctaacggtttcggtggtgtcttatctttcggtgtaaaagac**<br>**ttaccaaatgccgacaaggaaactgacccattcaaactttctggtgctca**<br>**agttgttgacaatttaaagcttgcctctaacttggccaatgttggtgatg**<br>**ccaagaccttagtcattgctccatacttcactacccacaaacaattaaat**<br>**gacaaagaaaagttggcatctggtgttaccaaggacttaattcgtgtctc**<br>**tgttggtatcgaatttattgatgacattattgcagacttccagcaatctt**<br>**ttgaaactgttttcgctggccaaaaaccatga**gtgtgcgtaatgagttgt	

	aaaattatgtataaacctactttctctcacaagtactatacttttataaa<br>acgaactttattgaaatgaatatcctttttttcccttgttacatgtcgtg<br>actcgtactttgaacctaaattgttctaacatcaaagaacagtgttaatt<br>cgcagtcgagaag	
HIS3red	tattgtgagggtcagttatttcatccagatataacccgagaggaaacttc<br>ttagcgtctgttttcgtaccataaggcagttcatgaggtatattttcgtt<br>attgaagcccagctcgtgaatgcttaatgctgctgaactggtgtccatgt<br>cgcctagCtacgcaatctccacaggctgcaaaggttttgtctcaagagca<br>atgttattgtgcaccccgtaattggtcaacaagtttaatctgtgcttgtc<br>caccagctctgtcgtaaccttcagttcatcgactatctgaagaaatttac<br>taggaatagtgCCATGGtacagcaaccgagaatggcaatttctactcggg<br>ttcagcaacgctgcataaacgctgttggtgccgtagacatattcgaagat<br>aggattatcattcataagtttcagagcaatgtccttattctggaacttgg<br>atttatggctcttttggtttaatttcgcctgattcttgatctcctttagc<br>ttctcgacgtgggcctttttcttgccatatggatccgctgcacggtcctg<br>ttccctagcatgtacgtgagcgtatttccttttaaaccacgacgctttgt<br>cttcattcaacgtttcccattgtttttttctactattgctttgctgtggg<br>aaaaacttatcgaaagatgacgactttttcttaattctcgttttaagagc<br>ttggtgagcgctaggagtcactgccaggtatcgtttgaacacggcattag<br>tcagggaagtcataacacagtcctttcccgcaatttcttttttctattac<br>tcttggcctcctctagtacactctatatttttttatgcctcggtaatgat<br>tttcattttttttttttccacctagcggatgactcttttttttcttagcg<br>attggcattatcacataatgaattatacattatataaagtaatgtgattt<br>cttcgaagaatatactaaaaaatgagcaggcaagataaacgaaggcaaag<br>**atgacagagcagaaagccctagtaaagcgtattacaaatgaaaccaagat**<br>**tcagattgcgatctctttaaagggtggtcccctagcgatagagcactcga**<br>**tcttcccagaaaaagaggcagaagcagtagcagaacaggccacacaatcg**<br>**caagtgattaacgtccacacaggtatagggtttctggaccatatgataca**<br>**tgctctggccaagcattccggctggtcgctaatcgttgagtgcattggtg**<br>**acttacacatagacgaccatcacaccactgaagactgcgggattgctctc**<br>**ggtcaagcttttaaagaggcGctaggggccgtgcgtggagtaaaaaggtt**<br>**tggatcaggatttgcgcctttggatgaggcactttccagagcggtggtag**<br>**atctttcgaacaggccgtacgcagttgtcgaacttggtttgcaaagggag**<br>**aaagtaggagatctctcttgcgagatgatcccgcattttcttgaaagctt**<br>**tgcagaggctagcagaattaccctccacgttgattgtctgcgaggcaaga**<br>**atgatcatcaccgtagtgagagtgcgttcaaggctcttgcggttgccata**<br>**agagaagccacctcgcccaatggtaccaacgatgttccctccaccaaagg**<br>**tgttcttatgtag**tgacaccgattatttaaagctgcagcatacgatatat<br>atacatgtgtatatatgtatacctatgaatgtcagtaagtatgtatacga<br>acagtatgatactgaagatgacaaggtaatgcatcattctatacgtgtca	

	ttctgaacgaggcgcgctttcctttttctttttgctttttcttttttt<br>tctcttgaactcga	
LEU2red	tcgaggagaacttctagtatatctacatacctaatattattgccttatta<br>aaaatggaatcccaacaattacatcaaaatccacattctcttcaaaatca<br>attgtcctgtacttccttgttcatgtgtgttcaaaaacgttatatttata<br>ggataattatactctatttctcaacaagtaattggttgtttggccgagcg<br>gtctaaggcgcctgattcaagaaatatcttgaccgcagttaactgtggga<br>atactcaggtatcgtaagatgcaagagttcgaatctcttagcaaccatta<br>tttttttcctcaacataacgagaacacacaggggcgctatcgcacagaat<br>caaattcgatgactggaaatttttttgttaatttcagaggtcgcctgacgc<br>atataccttttttcaactgaaaaattgggagaaaaaggaaaggtgagagcg<br>ccggaaccggcttttcatatagaatagagaagcgttcatgactaaatgct<br>tgcatcacaatacttgaagttgacaatattatttaaggacctattgtttt<br>ttccaataggtggttagcaatcgtcttactttctaacttttcttaccttt<br>tacatttcagcaatatatatatatatatttcaaggatataccattcta**at**<br>**gtctgcccctaagaagatcgtcgttttgccaggtgaccacgttggtcaag**<br>**aaatcacagccgaagccattaaggttcttaaagctatttctgatgttcgt**<br>**tccaatgtcaagttcgatttcgaaaatcatttaattggtggtgctgctat**<br>**cgatgctacaggtgttccacttccagatgaggcgctggaagcctccaaga**<br>**aggctgatgccgttttgttaggtgctgtgggtggtcctaaatggggtacc**<br>**ggtagtgttagacctgaacaaggtttactaaaaatccgtaaagaacttca**<br>**attgtacgccaacttaagaccatgtaactttgcatccgactctcttttag**<br>**acttatctccaatcaagccacaatttgctaaaggtactgacttcgttgtt**<br>**gtcagagaattagtgggaggtatttactttggtaagagaaaggaagacga**<br>**tggtgatggtgtcgcttgggatagtgaacaatacaccgttccagaagtgc**<br>**aaagaatcacaagaatggccgctttcatggccctacaacatgagccacca**<br>**ttgcctatttggtccttggataaagctaatgttttggcctcttcaagatt**<br>**atggagaaaaactgtggaggaaaccatcaagaacgaattccctacattga**<br>**aggttcaacatcaattgattgattctgccgccatgatcctagttaagaac**<br>**ccaacccacctaaatggtattataatcaccagcaacatgtttggtgatat**<br>**catctccgatgaagcctccgttatcccaggttccttgggtttgttgccat**<br>**ctgcgtccttggcctctttgccagacaagaacaccgcatttggtttgtac**<br>**gaaccatgccacggttctgctccagatttgccaaagaataaggtcaaccc**<br>**tatcgccactatcttgtctgctgcaatgatgttgaaattgtcattgaact**<br>**tgcctgaagaaggtaaggccattgaagatgcagttaaaaaggttttggat**<br>**gcaggtatcagaactggtgatttaggtggttccaacagtaccaccgaagt**<br>**cggtgatgctgtcgccgaagaagttaagaaaatccttgcttaa**aaagatt<br>ctctttttttatgatatttgtacataaactttataaatgaaattcataat<br>agaaacgacacgaaattacaaaatggaatatgttcatagggtagacgaaa<br>ctatatacgcaatctacatacatttatcaagaaggagaaaaaggaggatg	

	taaaggaatacaggtaagcaaattgatactaatggctcaacgtgataagg<br>aaaaagaattgcactttaacattaatattgacaaggaggagggcaccaca<br>caaaaagttaggtgtaacagaaaatcatgaaactatgattcctaatttat<br>atattggaggattttctaaaaaaaaaaaaaaatacaacaaataaaaaaca<br>ctcaatgacctgaccatttgatggagtttaagtcaataccttcttgaacc<br>atttcccataatggtgaaagttccctcaagaattttactctgtcagaaac<br>ggccttaacgacgtagtcgtcctcctcttcagtactaaatctaccaatac<br>caaatctgatggaagaatgggctaatgcatcatccttacccagcgcatgt<br>aaaacataagaaggttctagggaagcagatgtacaggctgaacccgagga<br>taatgcgatatcccttagtgccatcaataaagattctccttccacgtagg<br>cgaaagaaacgttaacacaccctggataacgatgatctggagatccgttc<br>aacgtggtatgttcagcggataatagacctttgactaatttatcggatag<br>tcttttgatgtgagcttgatcgttgtcaaattctttcttcatcaatctcg<br>cagcttcaccaaatcccgctaccaatgggggggccaaagtaccagatctt<br>aaacctctctcttggccaccaccggatagtaaaggttctaatctaactct<br>tggtctccttcttacatagatggcacctattccctttggaccgtaaatct<br>tgtgagaagaaattgatagtaaatcaatgttcatttcattgac	
URA3red	ttgaaatttttttgattcggtaatctccgaacagaaggaagaacgaagga<br>aggagcacagacttagattggtatatatacgcatatgtagtgttgaagaa<br>acatgaaattgcccagtattcttaacccaactgcacagaacaaaaacctg<br>caggaaacgaagataaatc**atgtcgaaagctacatataaggaacgtgctg**<br>**ctactcatcctagtcctgttgctgccaagctatttaatatcatgcacgaa**<br>**aagcaaacaaacttgtgtgcttcattggatgttcgtaccaccaaggaatt**<br>**actggagttagttgaagcattaggtcccaaaatttgtttactaaaaacac**<br>**atgtggatatcttgactgatttttccatggagggcacagttaagccgcta**<br>**aaggcattatccgccaagtacaattttttactcttcgaagacagaaaatt**<br>**tgctgacattggtaatacagtcaaattgcagtactctgcgggtgtataca**<br>**gaatagcagaatgggcagacattacgaatgcacacggtgtggtgggccca**<br>**ggtattgttagcggtttgaagcaggcggcagaagaagtaacaaaggaacc**<br>**tagaggccttttgatgttagcagaattgtcatgcaagggctccctatcta**<br>**ctggagaatatactaagggtactgttgacattgcgaagagcgacaaagat**<br>**tttgttatcggctttattgctcaaagagacatgggtggaagagatgaagg**<br>**ttacgattggttgattatgacacccggtgtgggtttagatgacaagggag**<br>**acgcattgggtcaacagtatagaaccgtggatgatgtggtctctacagga**<br>**tctgacattattattgttggaagaggactatttgcaaagggaagggatgc**<br>**taaggtagagggtgaacgttacagaaaagcaggctgggaagcatatttga**<br>**gaagatgcggccagcaaaactaa**aaaactgtattataagtaaatgcatgt<br>atactaaactcacaaattagagcttcaatttaattatatcagttattacc<br>cgcgaatctcggtcgtaatgatttctataatgacgaaaaaaaaaaaattg<br>gaaagaaaaagcttcatggcctttataaaaaggaactatccaatacctcg	

	ccagaaccaagtaacagtattttacggggcacaaatcaagaacaataaga<br>caggactgtaaagtaggacgcattgaactccaaagaacaacaagagttcc<br>aaaaagtagtggaacaaaagcaaatgaaggatttcatgcgtttgtactct<br>aatctggtagaaagatgtttcacagactgtgtcaatgacttcacaacatc<br>aaagctaaccaataaggaacaaacatgcatcatgaagtgctcagaaaagt<br>tcttgaagcatagcgaacgtgtagggcagcgtttccaagaacaaaacgct<br>gccttgggacaaggcttgggccgataaggtgtactggcgtatatatatct<br>aattatgtatctctggtgtagcccatttttagcatgtaaatataaagaga<br>aaccatatctaatctaaccaaatccaaacaaaattcaatagttactatcg<br>ctttttctttctgtatcgcaaataagtgaaaattaaaaagaaagatta<br>aattggaagttggatatgggctggaacagcagcagtaatcggtatcgggt<br>tcgccactaatgacgtcctacgattgcactcaacagaccttgacgctcac<br>gccgtagcgggcgacaagtcaaacggaacaaccgttgccgttcccatcgg<br>agtccga	
luxR (pACYCDuet-luxR-ymUkG1)	aaggagatatacc**atggaaaacatcaacgctgatgacacctacagaatca**<br>**tcaacaagattaaggcttgcaggtctaacaacgatattaaccagtgtttg**<br>**tccgacatgaccaagatggttcattgcgaatattacttgttggccatcat**<br>**ctacccacactctatggttaagtccgacatttccattttggacaactacc**<br>**caaaaaaatggcgtcagtattacgatgatgccaacttgattaagtacgac**<br>**ccaatcgttgattactccaactctaatcattctcccatcaactggaacat**<br>**cttcgaaaacaatgccgtcaacaaaaagtccccaaacgttatcaaagaag**<br>**ctaagacctctggtttgattaccggtttttctttcccaattcataccgct**<br>**aacaatggtttcggtatgttgtcttttgcccactctgaaaaggataacta**<br>**catcgactcattattcttgcatgcctgcatgaacattccattgatagttc**<br>**catctctggtcgataactataggaagattaacattgccaacaacaagtcc**<br>**aacaacgatttgaccaaaagggaaaaagaatgtttggcttgggcttgcga**<br>**aggtaaatcttcttgggatatttccaagattttgggttgctctgaaagaa**<br>**ccgttactttccatttgactaacgcccaaatgaagttgaacactaccaac**<br>**agatgccagtctatttctaaggctattttgaccggtgctattgattgtcc**<br>**atactttaagaattaa**ttaagtcgaacagaaagtaatcgtattgtaca	*NcoI*, *luxR*, *BsrGI*
plux-ymUkG1	**tcactgcccgctttccagtcgggaaacctgtcgtgccagctgcattaatg**<br>**aatcggccaacgcgcggggagaggcggtttgcgtattgggcgccagggtg**<br>**gtttttcttttcaccagtgagacgggcaacagctgattgcccttcaccgc**<br>**ctggccctgagagagttgcagcaagcggtccacgctggtttgccccagca**<br>**ggcgaaaatcc**TGTTTGATGGTGGTTCCTCTTCGCTATTACGCCAGCGGA<br>tcc*acctgtaggatcgtacaggt*ttacgcaagaaaatggtttgttatagt<br>cgaataaaTACTAGAGaaagaggagaaaTACTAGCC**atggtcagtgtcat**<br>**caaagaagaaatgaagatcaagttgcacatggaaggtaacgttaatggtc**<br>**atgcctttgttattgaaggtgatggtaaaggtaaaccatacgatggtact**<br>**caaactttgaacttgactgtcaaagaaggtgctccattgccattctctta**	*lacI*, *luxO+plux*, *Ribosome binding site*, *ymUkG1*, MluI

	**cgatattttgactaacgccttccaatacggtaatagagcttttactaagt**<br>**acccagccgatatcccagattactttaagcaaacttttccagaaggttac**<br>**tcctgggaaagaactatgtcttacgaagataacgctatctgcaacgtcag**<br>**atccgaaatttctatggaaggtgattgcttcatctacaagatcagattcg**<br>**atggtaagaactttccaccaaatggtccagtcatgcaaaaaaagactttg**<br>**aagtgggaaccatccaccgaaatgatgtatgttagagatggtttcttgat**<br>**gggtgatgtcaatatggctttgttgttggaaggtggtggtcatcatagat**<br>**gtgatttcaagacttcttacaaggccaagaaggttgttcaattgccagat**<br>**gctcataagatcgatcacagaatcgaaatcttgtcccacgatagagatta**<br>**ctccaaggttaagttgtacgaaaacgctgttgctagaaactctttgttgc**<br>**catctcaagcttctaagtaa**cgcgt	

All functional elements are indicated by lowercase letters. The underlined and bold sequences denote the restriction sites and protein-coding sequence, respectively. The italic lowercase letters denote the operator sequence (*tetO*, *phlO*, and *luxO*).

Supplementary Table 5. Repeat unit of synPs with multiple operators used in this study.

Operator	Repeat unit	Source
tetO7	TTTAC*tccctatcagtgatagaga*ACGTATGTCGAGTTTAC*tccctatcagtgatagaga*ACGATGTCGAGTTTAC*tccctatcagtgatagaga*ACGTATGTCGAGTTTAC*tccctatcagtgatagaga*ACGTATGTCGAGTTTAC*tccctatcagtgatagaga*ACGTATGTCGAGTTTA*tccctatcagtgatagaga*ACGTATGTCGAGTTTAC*tccctatcagtgatagaga*ACGTATGT	pTRE-tight (Clontech Laboratories)
phlO6	*atgatacgaaacgtaccgtatcgttaaggt*GA*atgatacgaaacgtaccgtatcgttaaggt*CA*atgatacgaaacgtaccgtatcgttaaggt*CA*atgatacgaaacgtaccgtatcgttaaggt*GT*atgatacgaaacgtaccgtatcgttaaggt*AG*atgatacgaaacgtaccgtatcgttaaggt*	pPhlF[A] (6)
luxO5	TTACGCAAGGTCGAACATAAGT*acctgtaggatcgtacaggt*TTACGCAAGGTCGAACATAAGT*acctgtaggatcgtacaggt*CGCTATTACGCCAGCGGATCC*acctgtaggatcgtacaggt*GA*acctgtaggatcgtacaggt*CA*acctgtaggatcgtacaggt*	This study

All functional elements are indicated by lowercase letters. Italicized letters denote the operator sequences (*tetO*, *phlO*, and *luxO*, respectively). The underlined sequence in the *luxO5* operator is derived from a previously reported sequence (7×*luxO)* (7).

Supplementary Table 6. Library size for each directed evolution experiment.

Genetic Switch	Randomized DNA	Estimated library size (Number of individual clones)	Related Figure
Tet-ON	rTetTA cassette	2×10^5	Figure 3
DAPG-OFF	PhlTA cassette	1×10^4	Figure 4B, S9A
Camphor-OFF	CamTA cassette	2×10^3	Figure 4C, S10
DAPG-ON (1st round)	PhlTA cassette	1×10^4	Figure 4D, S13A
DAPG-ON (2nd round)	rPhlTA cassette	Not determined	Figure 4D, S13C
HSL-ON (1st round)	LuxTA cassette	2×10^4	Figure 4F, S15A
HSL-ON (2nd round)	LuxTA cassette	Not determined	Figure 4F, S15B

References

1. Brachmann C. B., *et al.* (1998) Designer deletion strains derived from *Saccharomyces cerevisiae* S288C: a useful set of strains and plasmids for PCR-mediated gene disruption and other applications. *Yeast* 14(2):115–132.
2. Ishii J., *et al.* (2009) A simple and immediate method for simultaneously evaluating expression level and plasmid maintenance in yeast. *J Biochem* 145(6):701-708.
3. Ishii J., *et al.* (2014) Three gene expression vector sets for concurrently expressing multiple genes in *Saccharomyces cerevisiae*. *FEMS Yeast Res* 14(3):399-411.
4. Sivakumar S., Porter-Goff M., Patel P. K., Benoit K., & Rhind N. (2004) In vivo labeling of fission yeast DNA with thymidine and thymidine analogs. *Methods* 33(3):213–219.
5. Kaishima M., Ishii J., Matsuno T., Fukuda N., & Kondo A. (2016) Expression of varied GFPs in *Saccharomyces cerevisiae*: codon optimization yields stronger than expected expression and fluorescence intensity. *Sci Rep* 6:35932.
6. Stanton B. C., *et al.* (2014) Systematic transfer of prokaryotic sensors and circuits to mammalian cells. *ACS Synth Biol* 3(12):880-891.
7. Neddermann P., *et al.* (2003) A novel, inducible, eukaryotic gene expression system based on the quorum-sensing transcription factor TraR. *EMBO Rep* 4(2):159–165.
