# Supplementary material for: Robust and flexible platform for directed evolution of yeast genetic switches
Source: Nat Commun. 2021 Mar 23;12:1846. doi: 10.1038/s41467-021-22134-y (PMC7988172; doi:10.1038/s41467-021-22134-y)
Supplement: Supplementary file 2 — Description of Additional Supplementary Files [file 41467_2021_22134_MOESM2_ESM.docx]

**Description of Additional Supplementary Files**

**- File Name: Supplementary Data 1**

Description: Sequences for all primers used in this study for plasmid construction (Excel Spreadsheet).
