# Supplementary material for: Robust and flexible platform for directed evolution of yeast genetic switches
Source: Nat Commun. 2021 Mar 23;12:1846. doi: 10.1038/s41467-021-22134-y (PMC7988172; doi:10.1038/s41467-021-22134-y)
Supplement: Supplementary file 4 — Reporting Summary [file 41467_2021_22134_MOESM4_ESM.pdf]

nature research

Corresponding author(s): Jun ishii

Last updated by author(s): Feb 24, 2021

Reporting Summary

Nature Research wishes to improve the reproducibility of the work that we publish. This form provides structure for consistency and transparency in reporting. For further information on Nature Research policies, see Authors & Referees and the Editorial Policy Checklist .

Statistics

For all statistical analyses, confirm that the following items are present in the figure legend, table legend, main text, or Methods section.

n/a	Confirmed	
☐	☒	The exact sample size (*n*) for each experimental group/condition, given as a discrete number and unit of measurement
☐	☒	A statement on whether measurements were taken from distinct samples or whether the same sample was measured repeatedly
☒	☐	The statistical test(s) used AND whether they are one- or two-sided<br>*Only common tests should be described solely by name; describe more complex techniques in the Methods section.*
☒	☐	A description of all covariates tested
☒	☐	A description of any assumptions or corrections, such as tests of normality and adjustment for multiple comparisons
☐	☒	A full description of the statistical parameters including central tendency (e.g. means) or other basic estimates (e.g. regression coefficient) AND variation (e.g. standard deviation) or associated estimates of uncertainty (e.g. confidence intervals)
☒	☐	For null hypothesis testing, the test statistic (e.g. *F*, *t*, *r*) with confidence intervals, effect sizes, degrees of freedom and *P* value noted<br>*Give P values as exact values whenever suitable.*
☒	☐	For Bayesian analysis, information on the choice of priors and Markov chain Monte Carlo settings
☒	☐	For hierarchical and complex designs, identification of the appropriate level for tests and full reporting of outcomes
☒	☐	Estimates of effect sizes (e.g. Cohen's *d*, Pearson's *r*), indicating how they were calculated

Our web collection on statistics for biologists contains articles on many of the points above.

Software and code

Policy information about availability of computer code

Data collection	BD FACS Diva software (v5 and v8) and Beckman Coulter CytExpert (v2) were used to collect all of the flow cytometry data.
Data analysis	BD FlowJo software v.10 (BD Biosciences) was used to analyze the flow cytometry data.

For manuscripts utilizing custom algorithms or software that are central to the research but not yet described in published literature, software must be made available to editors/reviewers. We strongly encourage code deposition in a community repository (e.g. GitHub). See the Nature Research guidelines for submitting code & software for further information.

Data

Policy information about availability of data

All manuscripts must include a data availability statement. This statement should provide the following information, where applicable:

- Accession codes, unique identifiers, or web links for publicly available datasets
- A list of figures that have associated raw data
- A description of any restrictions on data availability

The source data underlying Figs. 2b, 3, 4b–d, and 4f and Supplementary Figs. 9a, 10, 12, 13, 15, and 17-19 are provided as a source data file. PDB files used in this study is available in Protein Data Bank (PDB) (ID: 2ZB9 and 1L3L). The following plasmid can be obtained from Addgene: pGK401red-rtetTAK8N, L131L (plasmid# 165968); pGK415-rtetTAK8N, L131L (plasmid# 165969); pGK415-phlTA2-1E (plasmid# 165970);; pGK415-luxTA2-4F (plasmid# 165971); pATP416-pluxO5-ptetO7-crtYBI (plasmid# 165975), pATP416-ptetO7-pphlO6-crtYBI (plasmid# 165976); pATP416-pluxO5-pphlO6-crtYBI (plasmid# 165977); pATP403red-hENT1 (plasmid# 166027); pGK401red-hENT1 (plasmid# 166028); pRS405red-phlTA2-1E (plasmid# 166029), pRS403red_luxTA2-4F (plasmid# 166030).

Field-specific reporting

Please select the one below that is the best fit for your research. If you are not sure, read the appropriate sections before making your selection.

- [x] Life sciences
- [ ] Behavioural & social sciences
- [ ] Ecological, evolutionary & environmental sciences

For a reference copy of the document with all sections, see nature.com/documents/nr-reporting-summary-flat.pdf

Life sciences study design

All studies must disclose on these points even when the disclosure is negative.

Sample size	No statistical methods were used to predetermine sample size. Sample size for all experiments in this study was determined to be adequate based on the magnitude and consistency of measurable differences between groups.
Data exclusions	No data were excluded from the analyses.
Replication	All attempts at replication were successful. We generally performed three independent experiments.
Randomization	Single clones from streaked plates were randomly picked when necessary.
Blinding	Blinding was not used in this study as bulk samples of microbial cells were used all experiments in this study.

Reporting for specific materials, systems and methods

We require information from authors about some types of materials, experimental systems and methods used in many studies. Here, indicate whether each material, system or method listed is relevant to your study. If you are not sure if a list item applies to your research, read the appropriate section before selecting a response.

Materials & experimental systems

n/a	Involved in the study	
[x]	[ ]	Antibodies
[x]	[ ]	Eukaryotic cell lines
[x]	[ ]	Palaeontology
[x]	[ ]	Animals and other organisms
[x]	[ ]	Human research participants
[x]	[ ]	Clinical data

Methods

n/a	Involved in the study	
[x]	[ ]	ChIP-seq
[ ]	[x]	Flow cytometry
[x]	[ ]	MRI-based neuroimaging

Flow Cytometry

Plots

Confirm that:

- [x] The axis labels state the marker and fluorochrome used (e.g. CD4-FITC).
- [x] The axis scales are clearly visible. Include numbers along axes only for bottom left plot of group (a 'group' is an analysis of identical markers).
- [x] All plots are contour plots with outliers or pseudocolor plots.
- [x] A numerical value for number of cells or percentage (with statistics) is provided.

Methodology

Sample preparation	Cell cultures were diluted to appropriate buffer or distilled water.
Instrument	BD FACSCantoII and Beckman Coulter CytoFLEX
Software	BD FACSDiva, Beckman Coulter CytExpert and BD FlowJo
Cell population abundance	10,000 cells or more
Gating strategy	Gating strategy was not used in this study.

- [ ] Tick this box to confirm that a figure exemplifying the gating strategy is provided in the Supplementary Information.
